# Seeing is Believing: Developing Multimodal Metabolic Insights at the Molecular Level

**DOI:** 10.1021/acscentsci.3c01438

**Published:** 2024-03-21

**Authors:** Rahuljeet S Chadha, Jason A. Guerrero, Lu Wei, Laura M. Sanchez

**Affiliations:** † Division of Chemistry and Chemical Engineering, California Institute of Technology, Pasadena, California 91125 United States; ‡ Department of Chemistry and Biochemistry, University of California, Santa Cruz, Santa Cruz, California 95064 United States

## Abstract

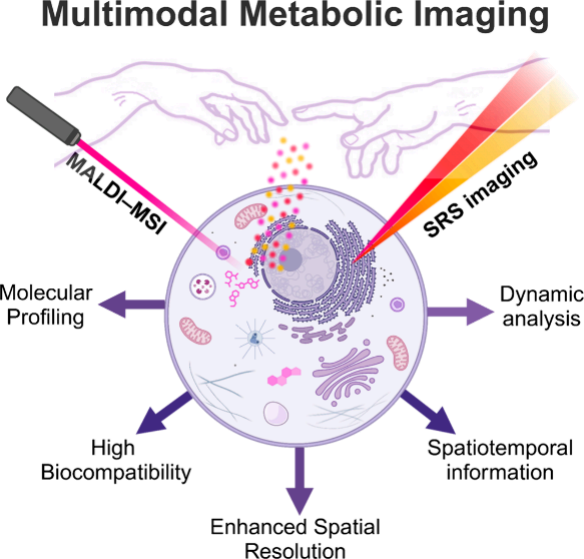

This outlook explores how two different molecular imaging approaches might be combined to gain insight into dynamic, subcellular metabolic processes. Specifically, we discuss how matrix-assisted laser desorption/ionization mass spectrometry imaging (MALDI-MSI) and stimulated Raman scattering (SRS) microscopy, which have significantly pushed the boundaries of imaging metabolic and metabolomic analyses in their own right, could be combined to create comprehensive molecular images. We first briefly summarize the recent advances for each technique. We then explore how one might overcome the inherent limitations of each individual method, by envisioning orthogonal and interchangeable workflows. Additionally, we delve into the potential benefits of adopting a complementary approach that combines both MSI and SRS spectro-microscopy for informing on specific chemical structures through functional-group-specific targets. Ultimately, by integrating the strengths of both imaging modalities, researchers can achieve a more comprehensive understanding of biological and chemical systems, enabling precise metabolic investigations. This synergistic approach holds substantial promise to expand our toolkit for studying metabolites in complex environments.

## Abstract 2

Integrating SRS microscopy and MALDI MSI offers a powerful imaging approach to investigate metabolites at the subcellular level in biological systems.

## I Introduction

Chemical imaging techniques have emerged as powerful tools for studying the heterogeneity observed in biological systems across different scales. Heterogeneity in chemical distribution throughout a biological system shapes development, function, physiology, and pathological responses.^1,2^ Among imaging methods, fluorescence microscopy is one of the most widely used tools owing to its high sensitivity, resolution, and selectivity.^3^ However, despite recent advancements in fluorescence imaging, this modality poses some key challenges for studying small biomolecules, e.g., metabolites, in living systems, especially systems that are not yet genetically tractable or lack a whole genome sequence for developing a reporter system.^4^ In the absence of reporter systems, challenges include the utilization and delivery of bulky fluorophores that can perturb the biological system, photobleaching, toxicity, and poor multiplexing due to broad fluorescent spectra causing spectral bleed-through.^5^

Emergent, orthogonal approaches for chemical imaging such as nonlinear Raman microscopy and mass spectrometry imaging (MSI) overcome some of these challenges and are quickly gaining popularity. In this outlook, we explore the benefits stemming from two distinct imaging techniques for metabolic and metabolomic analyses—SRS microscopy and MALDI-MSI, in the context of biological systems. First, we briefly introduce each methodology and provide an overview of metabolic applications to date. We then focus on the key advancements made in mapping the spatiotemporal dynamics of targeted (labeled) vs untargeted (unlabeled) metabolites in these fields. The key challenges in instrumentation, sample preparation, data interpretation, and processing as well as the practical considerations of using these complementary techniques are highlighted. Concluding our discussion, we offer perspectives on the capability of synergistically harnessing these two techniques for advancing metabolic analyses.

### SRS Microscopy

Raman spectromicroscopy probes the unique contrast from molecular vibrations and offers rich chemical information to gain insights into the composition and spatiotemporal distributions of biomolecules within heterogeneous biological systems.^6^ However, the conventional “spontaneous” Raman process has an inherently small transition cross-section. Hence, the microscopy suffers from low detection sensitivity with rather limited suitability for probing live cell dynamics. Additionally, interference from autofluorescence in biological samples also affects the Raman signal, which further hinders the analysis.

**Figure 1:**
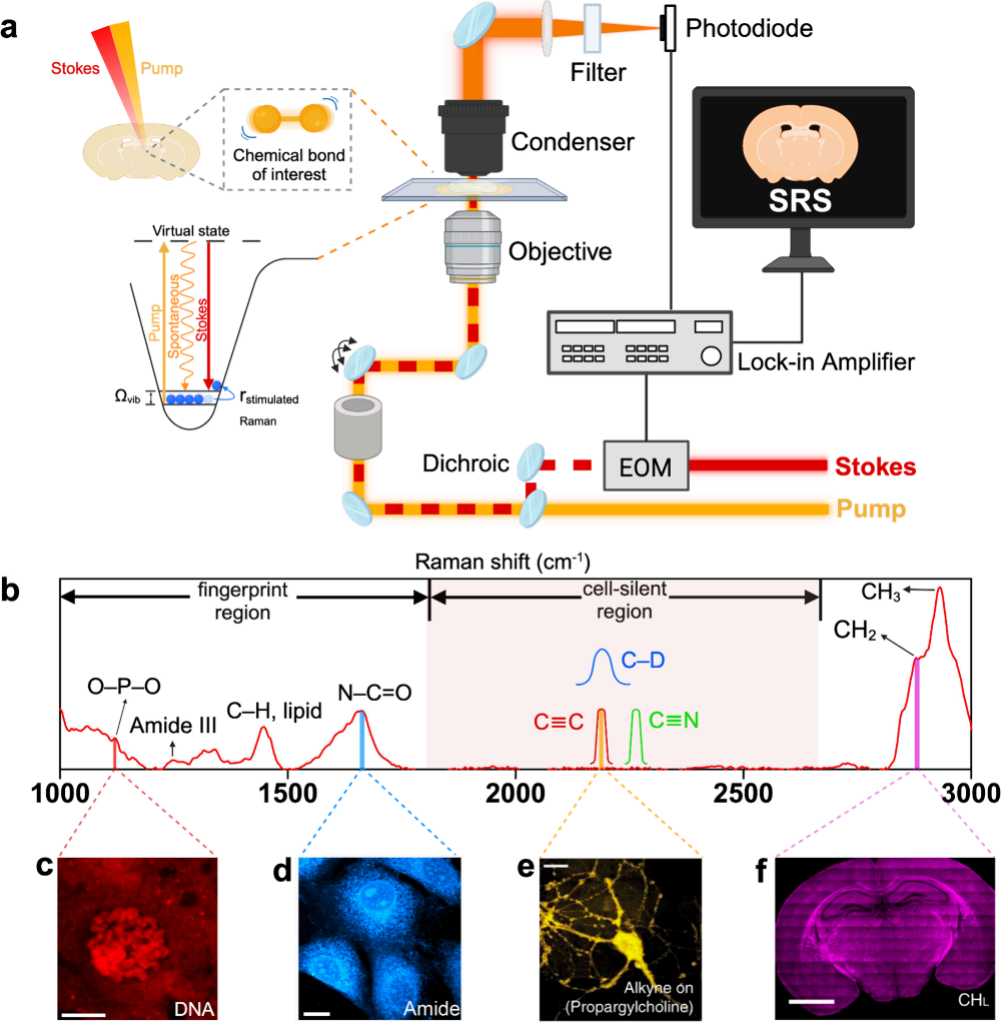
Illustration of the SRS microscope configuration and its diverse applications in different biological samples. (a) Left: Energy scheme of a vibrational mode from a chemical bond of interest probed by SRS spectro-microscopy. SRS is a nonparametric process that involves energy transfer from light to molecular vibration. When the energy difference between the pump and Stokes photons matches the vibrational frequency of the chemical bond of interest (for instance, 2845 cm^–1^ for CH_2_ stretching), the chemical bond is excited to a vibrational excited state. Right: SRS microscope setup. EOM = Electrooptic Modulator; created with BioRender.com. (b) Representative Raman spectrum of a human umbilical vein endothelial cell (HUVEC) illustrating the fingerprint region that spans from ∼600–1800 cm^–1^ and the cell-silent region from 1800–2800 cm^–1^ along with the high-frequency C–H region. (c) SRS image of a salivary gland cell from *Drosophila melanogaster* targeted at 1090 cm^–1^ in the fingerprint region (this peak originates from the symmetric dioxy-stretch of the phosphate backbone in nucleic acids); scale bar: 20 μm. Figure adapted with permission from X. Zhang et al.^31^ Copyright 2012 WILEY-VCH Verlag GmbH & Co. KGaA, Weinheim. (d) SRS image targeted at 1655 cm^–1^ (amide I band of proteins) in fixed NIH3T3 cells and (e) at 2142 cm^–1^ for choline metabolite imaging with 1 mM propargylcholine incubation in live neurons; scale bars: 10 μm. Figures adapted with permission from L. Wei et al.^14^ Copyright 2014, Springer Nature America, Inc. (f) Untargeted SRS imaging (mosaic) of lipids (CH_L_) from a whole brain tissue section of a mice pup. The images were acquired at 2845 cm^–1^ (CH_2_, lipids), decomposed, and mapped according to the unmixing procedure reported by Lu et al; scale bar: 2mm.^15^ Figure adapted with permission from L. Zhang et al.^32^ Copyright 2019, under exclusive license to Springer Nature Limited.

Recent efforts in the field of coherent Raman scattering (CRS) microscopy, including techniques such as coherent anti-Stokes Raman scattering (CARS) and stimulated Raman scattering (SRS), have successfully addressed many of these challenges. Specifically, in SRS microscopy, two spatially and temporally overlapping laser beams (pump and Stokes) excite the sample coincidently (Figure 1a). When the difference between the laser frequencies (Δω = ω_p_ – ω_s_) matches the vibrational frequency Ω of a specific chemical bond, a phenomenon called stimulated Raman excitation occurs. The signal is subsequently detected as intensity gain (Stokes) or intensity loss (pump). Conversely, when the frequency difference does not correspond to the targeted vibrational frequency, the SRS process does not take place. This results in a high degree of molecular specificity and selectivity with minimal nonresonance background. The utilization of near-infrared lasers makes SRS compatible with live cells with low photodamage, offers high spatial resolution in a diffraction-limited manner (∼400 nm in lateral; ∼1–2 μm in axial), and provides three-dimensional (3D) sectioning capability for tissue imaging.^7^ In low-scattering tissues, volumetric imaging achieves a depth of 300–500 μm, whereas in highly scattering tissues, such as brain tissue, the imaging depth is about 100 μm.^6^ However, recent SRS-tailored tissue clearing or expansion strategies have enabled imaging depth up to ∼1.1 mm and pushed the resolution down to sub-50 nm for either brain volumetric imaging or super-resolution investigations.^8−10^

In comparison to spontaneous Raman scattering, SRS provides an amplification of Raman scattering up to 10^8^–10^9^ times and allows for imaging at speeds more than 1000 times faster.^11^ SRS microscopy hence enables rapid molecular imaging at a video rate,^12,13^ making it well-suited for the study of dynamic cellular processes in live cells. For example, high-throughput SRS imaging of metabolites has been demonstrated in highly motile *E. gracilis* cells.^13^ Unlike CARS, SRS offers background-free chemical contrast, linear dependence on the analyte concentration, and higher detection sensitivity. As a result of these technical advancements, SRS microscopy has gained widespread recognition as one of the most powerful vibrational imaging modalities for quantitatively investigating metabolic processes.

By utilizing endogenous vibrational signatures or coupling with minimally perturbative and bioorthogonal vibrational probes including alkynes and isotopes, SRS can function as both an untargeted (unlabeled) and a targeted (labeled) approach across the wide Raman spectrum (Figure 1b). The labeled approach yields SRS a higher sensitivity, better molecule specificity, and expanded molecular resolving power. Moving forward, SRS could benefit significantly from the inclusion of more molecular species in single-cell metabolomics studies. Nonetheless, this versatile technique has already found abundant new opportunities for analyzing a wide range of metabolites (e.g., amino acids, fatty acids, nucleic acids, glucose, cholesterol, choline, glycogen, etc.) and uncovered many previously unknown regulatory and disease-related roles spanning from cell biology^14,15^ and cancer biology^16−18^ to neuroscience,^19−23^ microbiology,^24−26^ and even plant biology^27−30^ (Figure 1c–f, Table S1).

### MALDI-MSI

Mass spectrometry (MS) has the unique capability for detecting the accurate masses of diverse biomolecular complexes, small organic molecules, as well as individual atoms along with their respective isotopes.^33^ Since its inception, MS has undergone continuous technological advancements, particularly in terms of enhancing its mass resolving power, expanded mass range, and improved sensitivity. Specifically, we focus on matrix-assisted laser desorption/ionization mass spectrometry imaging (MALDI-MSI) as a label-free, soft ionization technique. By merging the sensitivity and high throughput capabilities of MS with spatial information, MSI enables the visualization and analysis of metabolites distributed within a biological sample.^34,35^ At present, MALDI-MSI has a diverse array of applications across research domains.^36−39^ MSI methods typically facilitate direct measurements of analytes from thin tissue sections, tissue or microbial surfaces, thinly sectioned organoids, or dried 3D cell cultures; in all instances, sample preparation is of utmost importance.^33,40,41^ Robust and reproducible sample preparation protocols ensure optimal ion efficiency and reliable spectral acquisition.

The ionization and desorption process for different biologically relevant metabolites in MALDI-MSI is highly reliant on the selection of the matrix. The matrix is typically composed of small, highly conjugated organic acids or bases that absorb UV energy. These matrices are cocrystallized with analytes, and during the rastering process, the laser irradiates the sample at designated spatial positions, which causes desorption and ionization from the sample surface. The resulting ions can then be separated in the mass analyzer and detected such that spectral data from every raster point is recorded. This data can be compiled into a single two-dimensional ion image,^37^ presenting a visual representation of biomolecules.^42−44^

**Figure 2:**
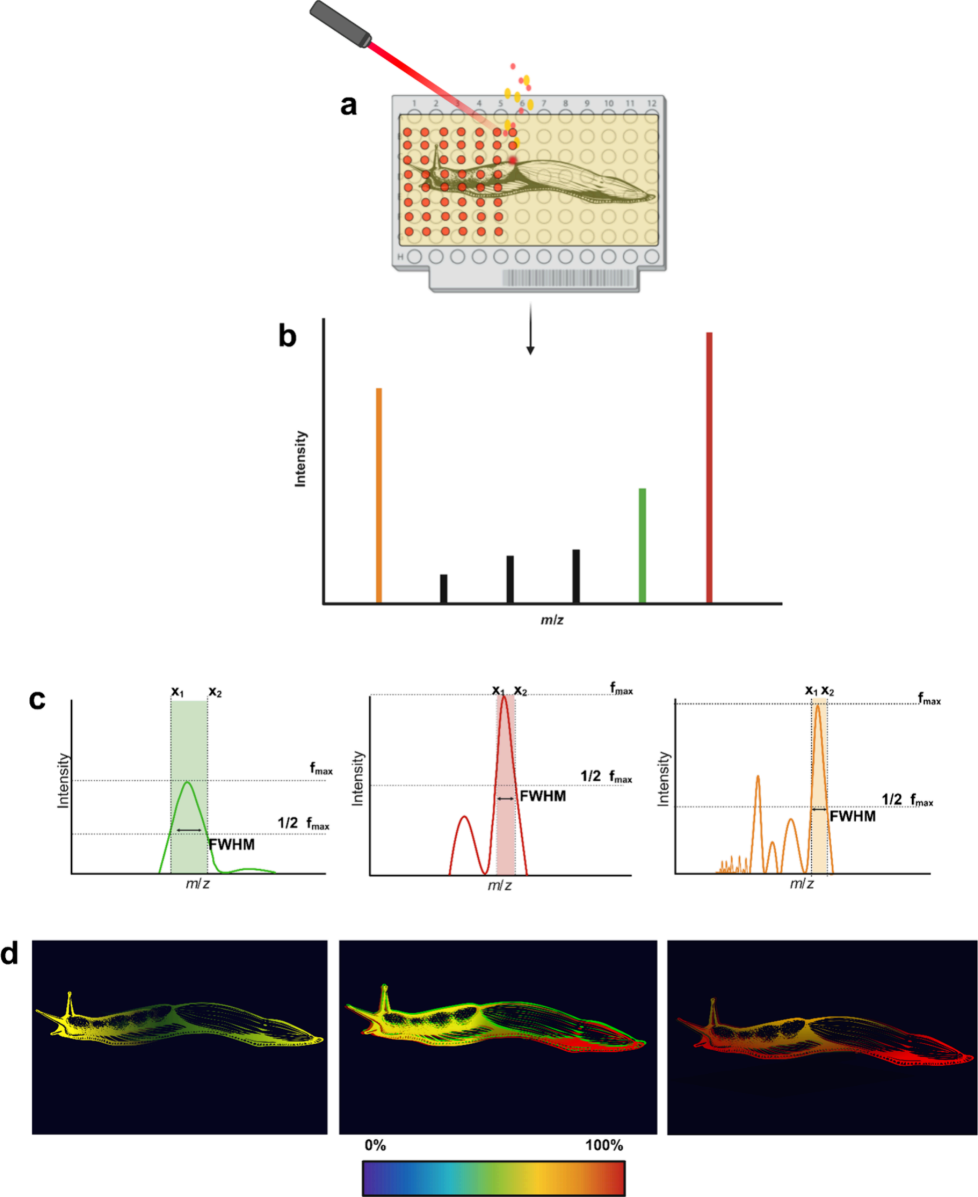
The spatial distribution of small molecules. Methods for preparing target-plate-bound tissues or samples enable them to withstand the vacuum environment within the source. (a) Adherence typically begins by placing a fresh-frozen thin tissue section onto a conductive surface such as a target plate (as depicted), where it is thaw-mounted and thus adhered to the plate. Samples that are grown in or on agar or agarose can be dried directly onto the conductive surface. In instances of charged decoupled sources, conductive surfaces are not needed. Following sample mounting, a matrix is then applied and allowed to cocrystallize with the analytes of interest within the sample, which facilitates the ionization process. This cocrystallization of the matrix and sample analytes is essential for the physical ablation (desorption) by the MALDI ionization laser source. (b) Subsequently, the ions are then separated within the mass analyzer and detected. (c) Resolution can be measured by assessing the full width of the peak at half its maximum height (FWHM). The resolution of spectral peaks is dictated by the specific type of mass analyzer employed. (d) Mass spectra are then compiled for each raster point and reconstructed into 2D ion images, providing visualization of the spatial distribution of small molecules. Created with BioRender.com.

Label-free spatial mapping has allowed for a number of unique discoveries for untargeted metabolomics work; however, MALDI-MSI also has unique challenges.^45−50^ For example, quantitative analysis of MSI data can be challenging because differences across the sample itself can cause interference and can lead to ion suppression.^51^ The presence of abundant ions may hinder the detection of low-abundance analytes. For MALDI specifically, surface height heterogeneity can result in areas where no spectral data was collected.^52^ The spatial resolution is limited for a number of reasons, including the size of the matrix crystals created via sublimation or spraying (<5 μm),^53,54^ but also by the stage movement (raster step) and spot size of the laser (5 μm-10 μm).^55^ It is worth noting that these numbers can be achieved with newer commercial mass spectrometers, while older instrumentation, which still have value and are widely used, will have lower spatial resolution capabilities. High spatial resolution imaging across a large area at high mass resolving power or coupled with ion mobility results in lengthy acquisition times and data processing, giving rise to data analysis and storage challenges.^56^ Despite some of these noted challenges, MALDI-MSI is a versatile tool for visualizing the spatial arrangement of ionizable metabolites within biological samples (Figure 2). Its widespread applications encompass various fields, including pharmacokinetics, biomarkers discovery, oncology research, and molecular profiling.^57−60^ Newer ion sources are currently being developed that enable 2 μm images, but are not widely commercially available.^61^

## II Sample Preparation and Envisioned Improvement

As is the case with any imaging modality, the primary steps to adopting new technology and techniques relate to sample preparation. Therefore, we will detail the sample preparation nuances for each method below with a focus on spatial resolution considerations as a short primer.

### SRS Microscopy

One of the key advantages of SRS microscopy is its minimal sample preparation requirements, enabling the analysis of live or fixed samples without the need for any specific pretreatments. The high specificity of the SRS signal makes it compatible with a wide range of biological samples, including standard tissue preparation methods such as fresh frozen tissues, paraformaldehyde-fixed tissues, and formalin-fixed paraffin-embedded (FFPE) tissues. In the case of paraffin, which causes an increase in background signals in the fingerprint region, samples can first be deparaffinized before imaging.^62^ Alternatively, targeted SRS imaging can be performed to eliminate the need for imaging the fingerprint region. Due to the noninvasive nature of SRS imaging, many live samples, including cells and organisms (such as *C. elegans*), can be retrieved after imaging, which allows performing longitudinal studies on the same sample. Additionally, most samples labeled with fluorophores can also be imaged in tandem with SRS microscopy with negligible crosstalk between the SRS signal and fluorescence. These properties underscore the high biocompatibility of SRS microscopy with different sample types.

For transmission-detection SRS imaging with optimal signals, samples are usually mounted between thin glass coverslips and glass slides using a spacer and imaging media (e.g., phosphate buffered saline, PBS) while avoiding air bubbles. Epi-detection SRS was also developed previously for nontransparent tissue imaging (e.g., live mouse brain), but at the sacrifice of signal levels.^12^ Proper sample hydration with the imaging media ensures heat dissipation and maintenance of optimum refractive indices during image acquisition.^63^

### MALDI-MSI

Sample preparation in MSI is critical for achieving optimal ion efficiency and acquiring high-quality spectral data (high mass accuracy and signal-to-noise, S/N). Sample preparation involves a multitude of considerations, encompassing not only the selection of the matrix but also the method employed for affixing samples to a substrate (e.g., stainless steel target plates, glass or ITO-coated slides, etc.), the sample’s thickness (especially for commercial instruments) and sample adherence (e.g., freeze–thaw mount, dry mounting to the substrate, double-sided conductive tape, etc.).

First, it is essential to carefully consider the analytes within the sample that are being evaluated to select the appropriate matrix (Table 1). The matrix dictates the types of analytes that can be desorbed and detected; the acidic or basic nature of the matrix favors negative or positive mode ionization. From our own experience, we have found that a number of representative molecule classes can be ionized using CHCA:DHB mixtures in positive mode. However, analytes such as lipids, which have acidic head groups, tend to ionize better in negative mode and remain difficult to detect with CHCA:DHB in positive mode. The generation of new matrices to selectively ionize specific compound classes and reactive matrices to target analytes with specific functional groups is a very active area of research. Table 1 summarizes common matrices and analyte classes.

**Table 1:**
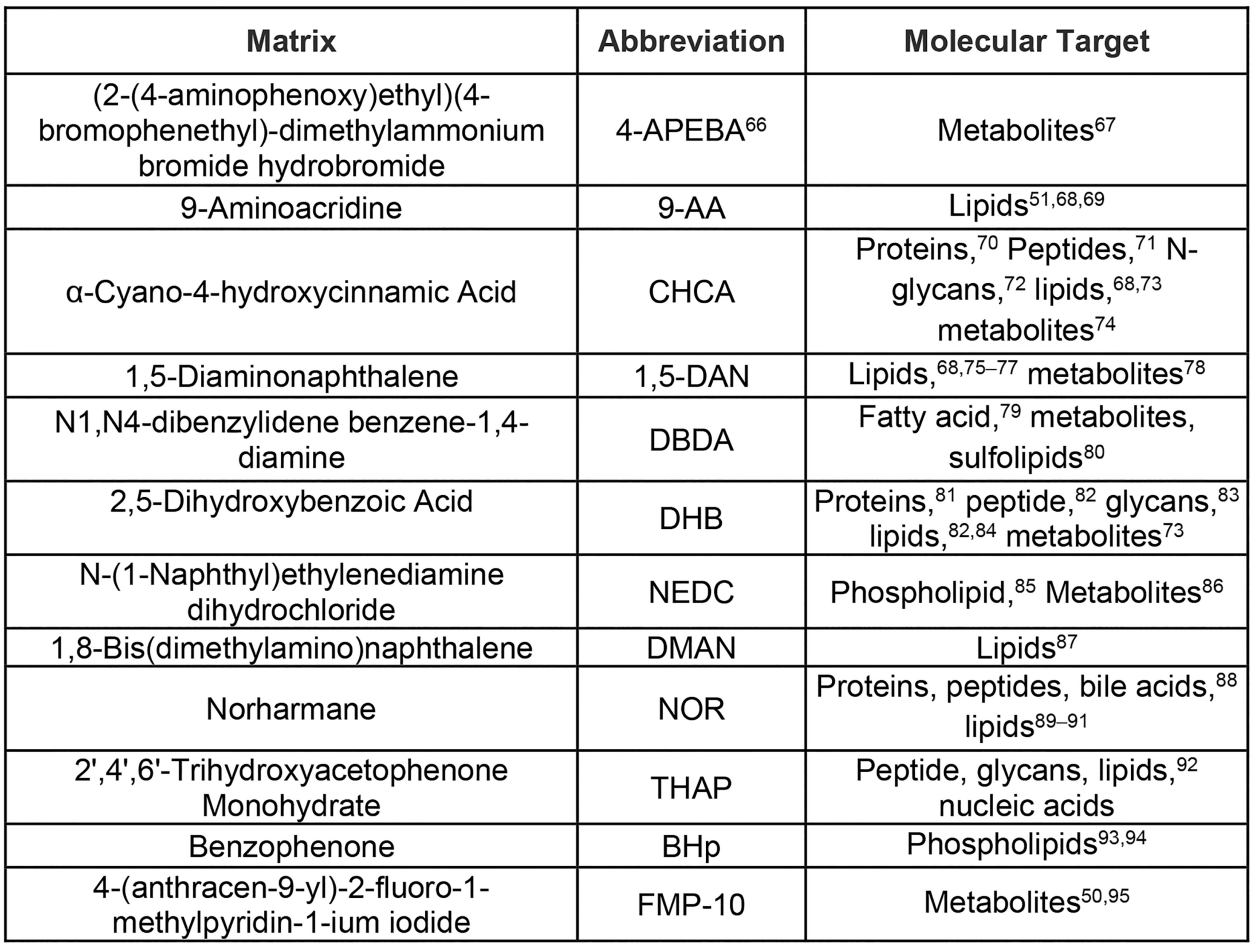
MALDI Matrices and Reactive Matrices^a^

a MALDI matrices are small, conjugated molecules that have UV-absorbing properties. These properties facilitate the ionization of biomolecules, such as proteins, peptides, carbohydrates, metabolites, and nucleic acids. The matrix tends to dictate the detection of the analyte of interest in different modes. Positive matrices often promote the ionization of analytes that contain functional groups that can accept protons, resulting in positively charged ions, while negative matrices tend to be nitrogenous bases that are prone to abstracting protons from analytes resulting in the formation of anions. Reactive matrices (L, M) can be used to react with specific functional groups on analytes of interest to form new compounds that are more easily ionized; they often result in the formation of inherently charged molecules. MALDI matrices are versatile for the ionization of biomolecules; hence, there is a significant overlap of usage of the matrices across biological classes.^66−95^

Once the matrix is selected, the matrix deposition should be optimized for the biological sample; the most common matrix deposition methods are direct spraying, sublimation, and sieving. Each of these methods generates different crystal sizes (ranging from 1 to 200 μm), which subsequently impacts the spatial resolution one is able to achieve.^53^ For instance, sublimation tends to generate the finest crystal size but is notoriously difficult to reproduce across biological replicates and typically requires researchers to devise strategies to confidently report the amount of matrix deposited to the sample, which typically involves weighing the target plate before and after sublimation to calculate the density of the matrix applied. While sieving is helpful for microbial samples, it is subject to nonhomogenous crystal formation, which can lead to inconsistent images.^64^ Regarding the sample itself, the sample’s height is important to account for. Disparities in sample height can lead to a decline in mass accuracy across the entire sample, introduce distortions in ion intensity, and give rise to localized zones commonly denoted as ‘dead spots’ in regions where the sample’s height lacks uniformity.^52^ Sample height heterogeneity can be challenging in commercial time-of-flight (TOF)-based mass analyzers, while other types of MSI modalities such as DESI do not suffer from this limitation.

Once the sample has been prepared, the desorption/ionization occurs as follows: a laser irradiates the sample, resulting in the physical ablation of its surface. Ionization occurs, and ions are separated in a mass analyzer before reaching a detector (Figure 2). A variety of mass analyzers are compatible with MALDI, such as QqTOF, TOF, FT-ICR, and the orbitrap to name a few, and these different mass analyzers have different mass resolution capabilities. The resolving power (RP) of a mass spectrometer, expressed as m/Δm, characterizes its ability to differentiate (resolve) ions with similar masses. A widely accepted standard for defining resolution involves Δm, which represents the full width of the peak at half its maximum height (FWHM). A lower RP results in broader-shaped peaks, whereas a higher RP yields narrower-shaped peaks, contributing to enhanced accuracy in mass measurement (Figure 2C). The repetitive laser pulses on the surface capture spectral data from each raster point, allowing for the data to be averaged and queried as pixels. These spectral data points can be compiled to construct a 2D ion image, facilitating the visualization of biomolecules distributed spatially across the sample (Figure 2). While this label-free approach is powerful, it is important to note that both physical constraints and instrumental factors constrain its application to single-cell analysis of subcellular components, particularly when trying to achieve a spatial resolution below 5–10 μm. Recently, a number of groups have drawn inspiration from ExM to push forward expansion mass spectrometry (ExMS), which seeks to overcome these constraints and increase the spatial resolution. Thus far, these ExMS approaches have successfully detected lipids in expanded tissue samples, although the extension to single cells and other metabolites has yet to be demonstrated.^53,65^

## III Single-Cell Metabolic Profiling Using (Un)targeted Imaging

Developing analytical tools to visualize metabolites at single-cell resolution is challenging due to their dynamic nature, structural diversity, turnover rates, and potential low abundance within limited sample volumes.^96^ In this section, we first discuss how researchers can leverage SRS microscopy as a promising technique to gain insights into metabolic dynamics at the single-cell level using untargeted and targeted approaches. For MSI, since analytes are resolved by measured accurate masses (Figure 2), we instead discuss the examples in which researchers have been able to measure analytes at high lateral resolutions to approach single-cell compatible measurements.

### SRS Untargeted Metabolic Imaging

Originally developed as a label-free modality, SRS microscopy facilitates highly specific, noninvasive imaging of endogenous molecules, encompassing various chemical bonds such as C–H, C=O, S=O, O–P–O, O–H, and amide which appear in the fingerprint region (600–1800 cm^–1^) and high-frequency C–H stretching region (2800–3100 cm^–1^) (Figure 1b). Untargeted metabolite SRS imaging has been applied to visualize various biomolecules such as proteins,^18^ lipids,^97−100^ nucleic acids,^15,31^ retinoids,^101^ cholesterol,^102^ cell walls (xylan),^103^ neurotransmitters,^22^ pharmaceutical compounds (PCs),^104^ and biofuels such as limonene and pinene^105−107^ in a variety of biospecimens (Table S1). Label-free SRS imaging has also been used for histopathology in the clinical setting to capture diagnostic hallmarks for diseases such as cancer.^18,108−114^

Despite the advantages associated with label-free imaging, SRS imaging of small molecules remains challenging owing to the presence of interference from other cellular components with overlapping Raman peaks in the crowded fingerprint region that limits its specificity. The use of chemometrics allows spectral deconvolution but adds significant complexity to data processing. Additionally, a label-free approach poses challenges for the visualization of dynamic metabolic processes such as the uptake, localization, and turnover of small biomolecules.^4^ Therefore, to improve chemical specificity, enhance detection contrast, and fully probe cellular dynamics, bioorthogonal vibrational tags have been introduced, extending detection beyond the realm of label-free SRS microscopy.

### SRS Targeted Metabolic Imaging

**Figure 3:**
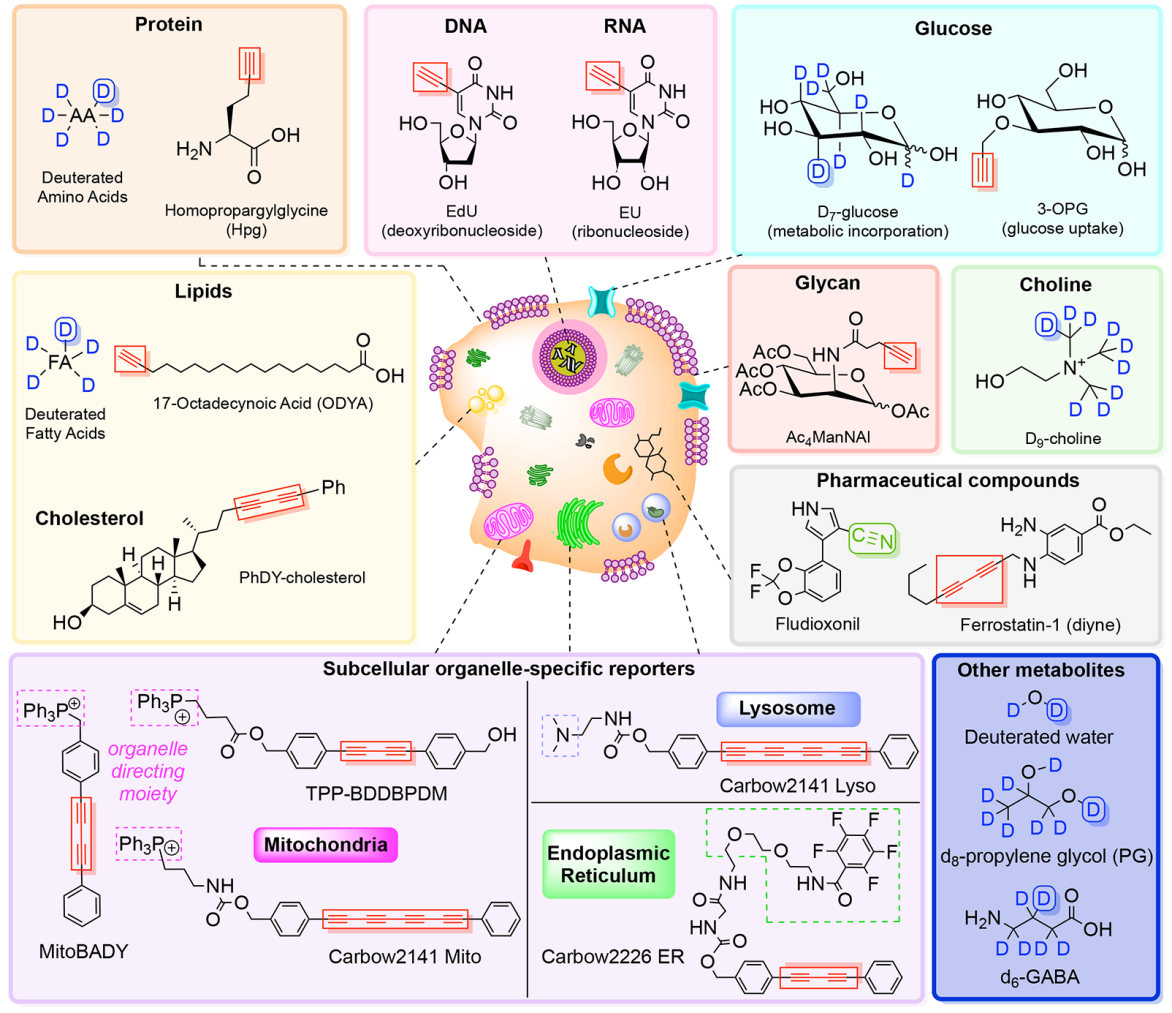
Stimulated Raman imaging of metabolites. Representative vibrational tags for targeted SRS imaging of metabolites such as lipids, glucose, choline, glycan, and more. An extended table is provided in the Supporting Information (Table S1).

Devoid of the endogenous peaks from biological samples, the cell-silent region (∼1800–2800 cm^–1^) offers a strategic opportunity to use vibrational tags (targeted SRS imaging) based on chemical bonds with vibrational frequencies that fall within this range. Using bioorthogonal Raman tags containing moieties such as alkynes (C≡C), nitriles (C≡N), and stable isotopes (such as deuterium and ^13^C) has not only enhanced sensitivity and specificity, but has also enabled visualization of spatiotemporal dynamics of small biomolecules, a capability not achieved through label-free SRS imaging. These metabolites include (but are not limited to) amino acids,^115^ fatty acids,^100,116,117^ sterols,^118,119^ glucose,^120−122^ glycan,^123^ choline,^124^ neurotransmitters,^125^ and pharmaceutical compounds such as ferrostatin^126^ and anisomycin^127^ (Figure 3 and Table S1).

In addition to tracking the uptake of stable-isotope labeled (SIL) metabolites, SRS imaging has been instrumental in observing their synthesis and turnover. For example, deuterated glucose has been coupled with SRS microscopy to visualize its full metabolism—from its catabolism and anabolic utilization to macromolecular synthesis in situ.^32^ This probe gets metabolically incorporated into different glucose-related biosynthesis pathways, resulting in distinct C–D Raman peaks that are influenced by the local chemical environment of the biomacromolecule. Through spectral unmixing, the different C–D spectra can be resolved into chemical maps of their corresponding biomacromolecules, enabling visualization of glucose metabolism. Furthermore, this strategy has enabled the visualization of the subcellular enrichment of glycogen, shedding light on the metabolic reprogramming of glycogen in cancer cells.^128^ In tandem with alkyne–glucose analogue (3-OPG) for assaying glucose uptake, two-color glucose imaging for simultaneous investigation of glucose uptake and metabolism, central to metabolic pathways, in live cells has been made possible.^121^

Apart from parallel phenotyping of multiple metabolic species, SRS imaging enables multiplexing of the same metabolic species through selective labeling of metabolic probes based on their structural and spectroscopic differences. For instance, Wei et al. achieved real-time visualization of the total complex proteome metabolism by employing a two-color pulse-chase imaging of proteins.^129^ Using two structurally different subsets of deuterated amino acids, proteins were labeled at different times, resulting in distinct Raman signals for multiplexed protein imaging. This approach could be extended to metabolites in dynamic processes by labeling different functional groups with stable isotopes. Similarly, isotopic labeling on alkynes and diynes has enabled the labeling of different metabolites, increasing the possibilities for multicolor SRS imaging.^130^ It is worth noting that any label other than a stable isotope may alter the biological function of a metabolite so it would be important to validate the altered metabolite prior to use in an imaging experiment. For instance, 3-OPG, which cannot enter the glycolysis pathway, serves as an effective probe to assay short-term glucose uptake. In contrast, deuterated glucose, which can be metabolically incorporated in cells, is a better probe for monitoring long-term glucose metabolism.

Organelle-targeted SRS reporters for subcellular imaging of cellular structures such as mitochondria,^131−136^ lysosomes,^136−139^ and the endoplasmic reticulum^136^ have also been developed. For instance, Bae et al. recently utilized an aryl-diyne-based Raman tag called TPP–BDDBPDM (Figure 3, Table S1) to develop a quantitative model to link mitochondrial membrane potential, a key indicator for mitochondria-mediated metabolic activities, with key pharmacokinetic properties (such as uptake rate and intracellular concentrations of TPP) in live cells.^132^ Taking advantage of the narrow line width of the Raman bands (<20 cm^–1^), Raman sensors for the detection of intracellular environments such as pH,^140^ ions,^141,142^ small signaling molecules such as hydrogen sulfide,^143^ as well as enzymatic activity^144^ have also been developed.

Enhancing SRS imaging sensitivity would allow more low-abundant metabolites to be detected. The use of these Raman tags has facilitated μM levels of sensitivity of metabolites. For example, the analytical sensitivity of the alkyne-containing thymidine analogue, 5-ethynl-2′-deoxyuridine (EdU) is 200 μM in live cells.^14^ Current efforts in the development of highly selective, sensitive, and multiplexed Raman palettes have further increased the detection sensitivity to the nM range. These include xanthene-based electronic preresonance enhanced Manhattan Raman Scattering (MARS) dyes and polyyne-based “Carbow” dyes.^136,145^ To expand the range of the current palette of Raman tags, novel metallacarborane probes that appear in the “unchartered territory” of the cell-silent window (2300–2800 cm^–1^) have also been recently reported.^146^ Ongoing advancements in the development of such Raman tags hold great promise for expanding the detection capabilities to include an increasing number of metabolites at subcellular resolution.

### MSI Single-Cell Imaging Applications

One of the main challenges associated with MALDI-MSI is its limited spatial resolution, which hampers its capacity to directly probe individual cells and their respective subcellular structures. The spatial resolution of MALDI-MSI is constrained by both instrumental and physical factors as discussed in Section II. Overcoming the spatial resolution limitations of MALDI-MSI remains an active area of research. For instance, Dunne et. al integrated MALDI-MSI with antibody-based single-cell spatial omics workflow, enabling the exploration of interactions within both cellular and extracellular matrix (ECM) domains in individual tissue sections.^147^ Their results highlighted the importance of considering the size of the region of interest when selecting an antibody-directed spatial technique. The combination of MALDI-immunohistochemistry (IHC) with fluorescence microscopy demonstrated its efficacy in achieving subcellular localization of small molecules, proving particularly advantageous for comprehensive scans of whole tissues.

Conversely, imaging mass cytometry (IMC), which relies on lanthanide-tagged antibodies and ICP MS, can attain high-dimensional subcellular resolution, effectively overcoming the multiplexed limitations observed in IHC. This study aimed to target the spatial information on single cells, specifically matrisomal N-glycan and ECM proteins. The overarching conclusion was that the optimal execution of single-cell omics with antibody detection occurred when MSI was performed on the same tissue, keeping the order of the complementary measurements in mind. Claes et. al illustrated the effectiveness of MALDI-IHC by investigating the synergy between untargeted on-tissue bottom-up spatial proteomics and targeted MALDI-IHC for examining distinctive markers within tumor microenvironments of breast cancer tissue.^148^ Zhang et. al employed a multimodal strategy for analyzing single cells across various cell lines, integrating trapped ion mobility (TIMS) with dual-polarity ionization MSI to conduct sequential data acquisitions on individual cells.^149^ This method enhances the coverage of the single-cell lipidome, enabling high-resolution spatial mapping of intracellular components and capturing lipidome heterogeneity between individual cells.

Rappez et al. address the challenge posed by MALDI single-cell metabolomics by developing SpaceM.^150^ SpaceM is an open-source solution that integrates light microscopy and MSI. This method enables the acquisition of in situ metabolic profiles for individual cells by leveraging segmented microscopy images so that precise cell identification occurs, allowing for the quantification of spatial arrangement by morphometric properties and fluorescence intensity. Cells then undergo MSI for metabolite annotation, which provides insight into their metabolomic profiles before normalization. Precise MALDI pixel registration and single-cell intensity normalization are conducted to artificially map metabolites onto single cells, although the metabolite annotations rely on high-resolution MS^1^ measurements, which results in level 4 identifications.^151^ While there have been numerous efforts to enhance single-cell imaging in lipidomics and proteomics investigations, there is still currently a notable absence of high specificity toward the context of metabolomic studies.

## IV Multimodal Workflow for Metabolic Imaging

The integration of multimodal techniques, specifically combining SRS with MSI, represents a robust approach for investigating metabolic processes and elucidating disease biomarkers. We envision how specific studies that our laboratories have conducted might have been enhanced by applying a multimodal workflow.

**Figure 4:**
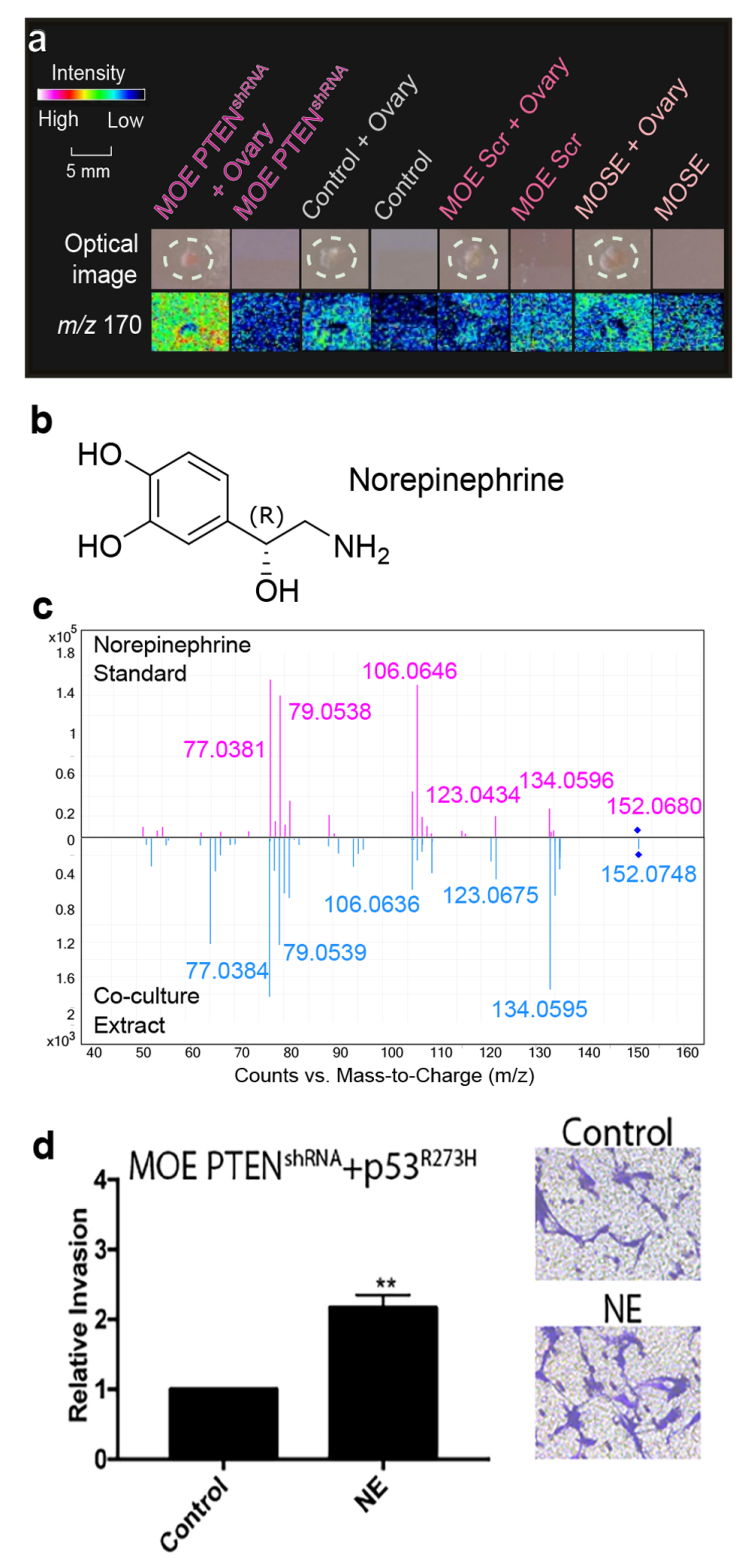
Mass spectrometry imaging was utilized to identify norepinephrine in high-grade serous ovarian cancer 3D cell cultures. (a) A 3D coculture protocol was developed to image FTE cells and murine ovaries. 3D agarose plugs were placed on a slide with a MOE PTEN^shRNA^ mutation cell line, and MOE Scr^shRNA^, a wildtype cell line, was used to ensure that the *m*/*z* signals being detected in HGSOC are specific to the MOE PTEN^shRNA^ cell line. MOSE cell lines were used as a control to verify that signals are specific to the MOE cell lines. *m*/*z* 170 was produced in higher abundance in the MOE PTEN^shRNA^ coculture condition. (b) The structure *m*/*z* 170 was found to be norepinephrine. (c) Tandem mass spectrometry analysis of the norepinephrine standard and the coculture extract was used to confirm the structural assignment. (d) Norepinephrine induced a 2-fold increase in the invasion of MOE p53^R273H^ + PTEN^shRNA^ cells, thereby substantiating the involvement of mutant p53 in the heightened invasive response to norepinephrine in fallopian-tube-epithelium-derived HGSOC. Reproduced with permission from ref (45). Copyright 2018 American Chemical Society.

In a study by Zink et al., we employed MSI to unveil the chemical crosstalk between tumorigenic fallopian tube epithelial cells (FTE) and a healthy ovary, which models the initial metastasis event in high-grade serous ovarian cancer (HGSOC) development (Figure 4).^45^ Briefly, our experimental methodology involved using a 3D coculture of murine tissue and FTE cells. An 8-well chamber, affixed to the center of an ITO slide, was prepared with 4 different cell lines to control for cell and gene specificity. The entire experiment took 4 days for incubation followed by a day for MS sample preparation and analysis. However, certain considerations arise. Are the selected cell lines appropriate, and is a 4-day incubation period sufficient? Given the limitations of the size of the growth chambers, it would be beneficial to have screened multiple FTE cell lines that have different mutations commonly observed in HGSOC or cell lines derived from patients rather than murine-derived FTE. Additionally, it is unclear if a 4-day incubation time was the most ideal. If we envision the rapid screening and live cell compatibility that SRS offers, we could have made more informed decisions about which cells to use for these experiments and which day of the coculture provides the most unique chemical signatures via SRS. This would ensure that the biological conditions are screened over multiple conditions while providing the most chemically rich information.

In the high spatial-resolution regime, SRS imaging can be utilized to conduct metabolic investigations not only at the global level but also down to the subcellular level. For instance, in a recent paper by Du et al, we demonstrated the use of stimulated Raman spectro-microscopy for the identification of phenotype-dependent metabolic susceptibilities in patient-derived, BRAF mutant melanoma lines with varying levels of differentiation.^152^ Mutations in the BRAF gene are known to cause elevated kinase activity, and this gene has been identified as an oncogene in malignant melanoma.^153^ At the global level, we identified the fatty acid synthesis pathway as a pharmacological target for differentiated melanocytic cells.

**Figure 5:**
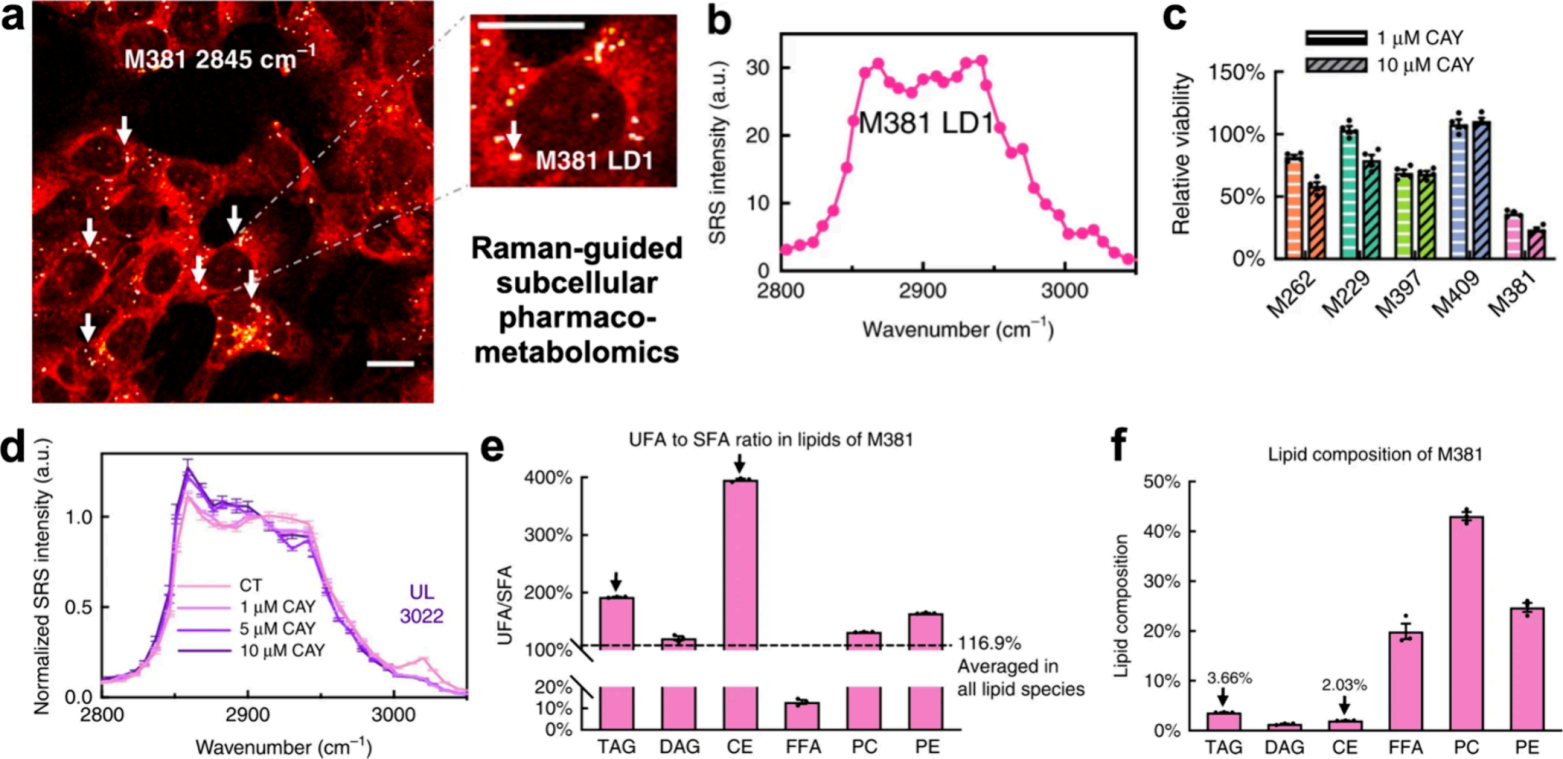
SRS microscopy of lipid droplets (LDs) reveals a unique metabolic susceptibility of monounsaturation in M381 cells. (a) Label-free SRS imaging of lipids (−CH_2_ channel, 2845 cm^–1^) for M381 cells. The arrow in the zoomed-in picture depicts LD (marked as M381 LD1). Scale bars, 20 μm. (b) A representative hyperspectral SRS (hSRS) spectrum from the LD (in a) at the high-frequency C–H stretching region (2800–3050 cm^–1^); (c) Relative viability of five cell lines after 3 days of treatment with 1 μM and 10 μM CAY10566 (CAY), an SCD1 inhibitor. Inhibition of MUFA synthesis by blocking SCD1 leads to a significant decrease in viability in the M381 cell line as compared to the other four cell lines. (d) hSRS spectra (normalized to 2908 cm^–1^) from LDs of M381 cells treated without (CT) or with serial concentrations of CAY10566 for 3 days. The peak at 3022 cm^–1^ represents unsaturated lipids (UL) while the broad band from 2950 to 3000 cm^–1^ represents cholesteryl ester (CE). Inhibition of SCD1 with CAY10566 leads to a decrease in UL and CE in LDs of M381 cells. (e) Average unsaturated fatty acid (UFA) to saturated fatty acid (SFA) ratio in each lipid class of M381 cells (*n* = 3 biological trials) obtained through lipidomics. The LD-enriched lipid species, triacylglycerides (TAG), and CE (both marked with black arrows) had the highest UFA:SFA. (f) Percentage lipid composition of each major lipid class of M381 cells (*n* = 3 biological trials). Bulk lipidomics data shows that the LD-enriched species (in e) are averaged out and only constitute a total of <6% of the total lipids in M381 cells. Therefore, there is an increased level of unsaturation in the intracellular LDs of M381 cells, suggesting a novel lipid regulation process in this mesenchymal cell line. Data is shown as mean ± SEM. Figure adapted with permission from Du et al.^152^ under Creative Commons license. Copyright 2020, Springer Nature.

At the subcellular level, SRS microscopy revealed a unique druggable metabolic susceptibility of lipid monounsaturation within the intracellular lipid droplets (LD) of dedifferentiated mesenchymal cells with innate resistance to BRAF inhibition (M381 cells, Figure 5a,b). The hyperspectral SRS (hSRS) data revealed that inhibition of stearoyl-CoA desaturase-1 (SCD1 or Δ9-desaturase), the rate-limiting enzyme for monounsaturated fatty acid (MUFA) synthesis from saturated fatty acid (SFA), led to a decrease in unsaturated lipids in LD, which was followed by the formation of intracellular phase-separated solid membrane (SM) domain and, ultimately, cellular apoptosis (Figure 5c,d). It is worth noting that this metabolic susceptibility was not detected through bulk metabolomics or transcriptional analysis but only through unique subcellular SRS analysis, emphasizing the role of subcellular heterogeneity. However, information-rich lipidomics via LC-MS/MS played a key role in validating and further interpreting our spatially resolved hSRS measurements—it provided key mechanistic insights into the disruption of lipid homeostasis and played a pivotal role in pinpointing the specific metabolic regulation pathway (Figure 5e,f). If the sample could have been directly analyzed via MSI after SRS imaging, the lipidome at specific spatial locations could have been immediately resolved by using ion pairing and electron-induced dissociation to characterize lipid structures.^154,155^ Such an integrated approach using MALDI–MSI and SRS microscopy could likely provide new avenues to directly identify metabolites within regions of interest.

**Figure 6:**
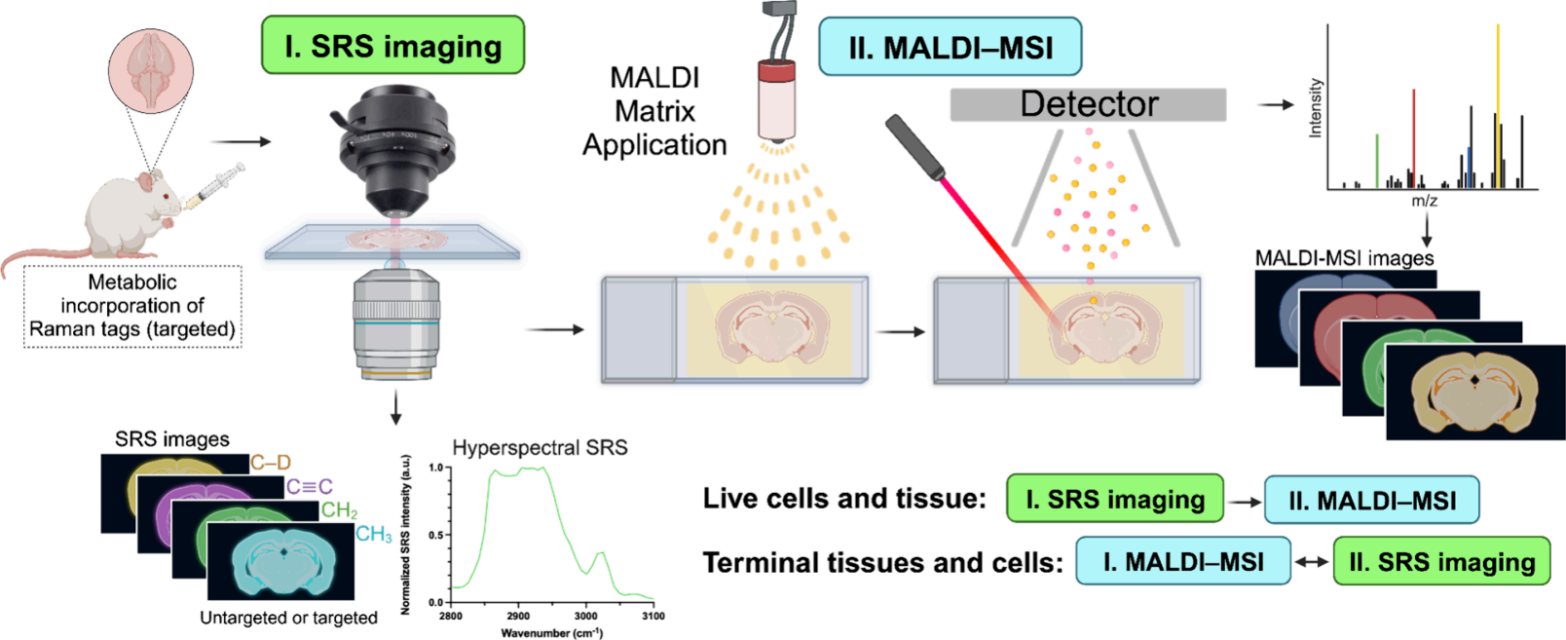
Hypothetical Multimodal SRS–MALDI-MSI workflow. Proposed integrated platform for multimodal imaging using SRS microscopy and MALDI–MSI for biospecimen. Created with BioRender.com.

In the context of multimodal imaging, a flexible workflow can be envisioned for the simultaneous metabolic imaging of the same tissue section (Figure 6). Consider a scenario in which live tissues are being used to analyze the metabolites within a sample. In this case, SRS imaging can be performed as the initial step to noninvasively visualize the presence of metabolites at the global level. SRS imaging can also guide the selection of the appropriate MALDI matrix for the subsequent MALDI–MSI metabolic analysis based on the stretches for specific functional groups that could be observed via SRS imaging. This preliminary SRS data can then facilitate a more detailed exploration of the specific metabolites of interest in live samples. For instance, SRS imaging could be leveraged to noninvasively and selectively map out the lipids present in the samples. The targeted SRS approach could also be integrated to assay the turnover of potential fatty acids of interest (e.g., using deuterated oleic acid as targeted unsaturated lipids) to comprehensively capture its time-dependent metabolic regulation. While hSRS can add layers of information on certain molecular species of saturated or unsaturated lipids (UL), and cholesteryl esters (CE), its full molecular scope is constrained in this regard. In such instances, MALDI-MSI can provide the missing information on the types and classes of lipids present in the sample.

MALDI-MSI has the capacity to specifically identify metabolites and lipids in an untargeted manner. The incorporation of ion pairing agents and different forms of dissociation techniques has facilitated the structure elucidation of lipids.^154,155^ Ion mobility spectrometry (IMS) has proven to be a valuable enhancement to MALDI-MSI, specifically facilitating the separation of isomeric structures based on their 3D structure. Collision cross sections are becoming popular as a new chemical property metric to aid in the identification of known unknowns. The degree of separation achieved by IMS, the fragmentation of particular ions by MS/MS, coupled with SRS, enhances specificity, facilitating the identification of molecules pivotal in cellular function and biological pathways undergoing alteration in abnormal states. It is also worth noting that MSI may be a more sensitive detection method for specific metabolites that have high ionization efficiencies, such as those at nM levels.

In cases of fixed/terminal tissues, there is greater flexibility in performing either modality first (Figure 6). As reported in a recent RaMALDI platform,^156^ certain MALDI matrices such as DAN, if applied to the sample, preserve the Raman signal quality and integrity as they do not cause interfering background Raman signals. Therefore, the application of certain matrices before SRS imaging should not hamper Raman analysis.

### Data Processing, Databases, and Data Accessibility

A major consideration for future multimodal integration is how one might merge data types from experiments with different spatial resolutions. It is important to consider the availability of open-source data file formats, data analysis options, and databases for subsequent metabolite annotation. For instance, in MSI, the field has converged on imzML as an open-source file format.^157^ Raw data files from vendors proprietary formats can be converted to imzML so that data can be opened and analyzed in open source programs such as Cardinal,^157,158^ MSiReader,^159^ or METASPACE.^160^ While vendor-proprietary software has greatly enhanced our ability to analyze data sets with statistical and annotation capabilities, it is often prohibitively expensive in the academic setting, and the number of installations is often limited, which further prevents researchers at various career stages from accessing and analyzing the data. Accompanying data analysis can be the access to the data itself. Deposition of the raw and converted data, as well as the associated metadata, is critical in facilitating the development of new data processing and analysis workflows; examples of these repositories include Metabolights, Metabolomics Workbench, and METASPACE.^160−162^ Additionally, information learned across data sets can be equally as valuable when thinking about the implementation of artificial intelligence to multimodal data sets. A recent major success story was the creation of AlphaFold,^163^ which leveraged crystallography data from the Protein Data Bank^164^ to train the model to predict structures of proteins that do not have any available crystal structures. Many times, it is not yet clear what we may be able to learn from the data in repositories, but the creation of new tools and an the increase in computational power is sure to aid our ability to interpret multimodal data sets.

In the Raman field, one of the obstacles that currently remains is the lack of comprehensive open-access databases for biologically relevant molecules such as metabolites and pharmaceutical compounds, comparable to those available for MSI. These limitations in data accessibility pose additional challenges in consistent peak identification and characterization, particularly in the fingerprint region, which becomes more pronounced if the target analyte is unknown. More importantly, in hydrated environments such as biological systems, Raman peaks can differ from their counterparts in solid systems due to factors such as peak broadening or peak shifting which add further complexity to data interpretation. Therefore, well-annotated datasets incorporating these variations should assist in conducting accurate data analysis as well as refinement of machine learning (ML) algorithms for biological investigations. In addition to Raman spectra, depositing raw images should also facilitate the benchmarking comparison across laboratories, and benefit the broader analysis by leveraging the development of robust ML algorithms.

Since detected Raman signals may vary with different instrumental configurations, even when using the same samples, concerted efforts are required to increase the sharing of standardized protocols and standardization procedures within the (stimulated) Raman community. These practices could contribute to mitigating these differences and building computational models that are tolerant of these spectral variations.^165^ Open-source data processing approaches can also facilitate the comparability of data acquired from diverse biosystems by different researchers, allowing for easier comparison and analysis.^166^ Hence, encouraging researchers to actively participate in data-sharing initiatives, creating open-source tools, and establishing common standards would culminate in a more unified approach to overcoming these challenges similar to what has been seen in the MSI field, vide supra.

Present collaborative initiatives are also underway to establish a standardized data format across different imaging fields. For instance, an open-source converter called Raman2imzML that transforms Raman data into the imzML format has been recently reported.^167^ Joint efforts such as these can further streamline data processing across different imaging communities, facilitating the advancement of multimodal metabolic imaging.

## V Laser-Focused Insights into Future Imaging

While a few studies have explored the combination of Raman microscopy and MSI,^156,168−170^ to the best of our knowledge, there is a scarcity of literature on the combined use of SRS microscopy and MALDI-MSI for multimodal imaging. In this section, we envision various practical scenarios in which the complementarity of both modalities can be employed within a multimodal workflow to inform researchers on metabolic discoveries:

1) **Cancer:** For cancer tissues, SRS imaging can noninvasively and rapidly provide information on lipid phenotyping, distribution, desaturation levels, and cellular morphology.^109,171^ This information can be leveraged for MSI by guiding researchers to specific regions of interest for data acquisition such that high mass resolution experiments with ion pairing, MS/MS, or ion mobility can be utilized to further characterize the lipids to identify the lipid chains, double bond positions, and detect additional classes such as sphingolipids, phospholipids, and glycerolipids that may not be easily detected with SRS imaging, enabling a more comprehensive understanding of tumor heterogeneity and progression in such tissues.^172^ This multimodal approach would save time while also leading to direct identification of lipid species.
2) **Clinical (tissue biopsy) samples:** This versatile approach can be extrapolated to clinical scenarios involving tissue biopsy samples. SRS imaging facilitates rapid, label-free visualization of tissue, characterized by its unique capacity for chemical contrast, enabling the discernment of alterations such as protein-to-lipid ratio within tumor-infiltrated tissue and variations in cellularity.^114^ It can also be used for tissue histology due to the chemical contrast of intrinsic metabolites.^173^ MALDI-MSI offers comprehensive molecular insights across a wide mass spectrum, spanning small molecules to large proteins,^174,175^ by generating detailed 2D ion images of specific compounds that may have otherwise not been detected in the fingerprint region of the SRS imaging. The capacity to correlate chemotypes in tissue regions through MSI renders it valuable for investigating disease progression, discovering biomarkers, and enhancing diagnostic precision.
3) **Neuroscience:** SRS microscopy can perform real-time imaging of dynamic processes such as (de)myelination in single cells,^176^ as well as the visualization of small molecules such as glucose or neurotransmitters.^22,125^ It can generate high spatial-resolution quantification maps of intracellular, pathophysiological protein aggregates.^23,177^ On the other hand, MALDI-MSI can assist in detailed lipid and metabolic profiling at different time points during neurological processes.^178^ Furthermore, if one employs a reactive MALDI matrix, such as FMP10, MSI can pinpoint neurotransmitters, neuropeptides, and other neurochemicals that are involved in these processes that have not been resolved without the use of stable isotopes by SRS.^50,179^ This multimodal approach can provide insights into the pathways implicated in neurodegenerative diseases.
4) **Microbiome Analysis:** SRS imaging allows the observation of microbial cells and their structural elements in a spatiotemporal context.^25,180,181^ This capability enables the examination of alterations in both the spatial distribution and chemical composition of microbial colonies over time. However, the majority of microbial chemistry remains uncharacterized, and many genomes are still not sequenced, hindering reliable predictions of microbial products.^182^ This gap in knowledge of which microbes produce which specialized metabolites may make the SRS data difficult to fully interpret. MALDI-MSI can be used to gather untargeted data from specific regions of interest which help aid research efforts toward identification of the microbial molecules of interest.
5) **Pharmaceutical screening and drug discovery:** SRS can be used for monitoring the uptake, delivery, and localization of pharmaceutical compounds (PCs).^104^ Targeted SRS imaging can further enable longitudinal analysis of PCs in biosystems.^127^ Additionally, MALDI-MSI can identify the molecular changes induced by these PCs in biological systems.^183^ When heavy labeled versions of the PCs are available, absolute quantification can be achieved with MSI.^184^ This can provide valuable information on drug uptake, localization, pharmacokinetics, and the identification of druggable targets in biological systems.

Combining these two techniques can contribute essential pieces of data to fill in the gaps in the metabolic puzzle. The integration of these synergistic modalities—SRS microscopy and MALDI-MSI—can offer comprehensive metabolic profiling, enhanced spatial resolution, and deeper insights into molecular mechanisms, paving the way for metabolic discoveries in biology and medicine. We strongly urge both fields to move toward FAIR data practices so that data can continue to be reused and learned from as computational approaches are developed.^185^

## Supporting Information Available

List of metabolites for stimulated Raman imaging of untargeted (unlabeled) or targeted (labeled using biorthogonal tags) along with their Raman shift (cm^–1^) and vibrational contrast; list of acronyms and nonstandard abbreviations (PDF)
Transparent Peer Review report available (PDF)
