# Supplementary material for: Seeing is Believing: Developing Multimodal Metabolic Insights at the Molecular Level
Source: ACS Cent Sci. 2024 Mar 21;10(4):758–74. doi: 10.1021/acscentsci.3c01438 (PMC11046475; doi:10.1021/acscentsci.3c01438)
Supplement: Supplementary file 1 — oc3c01438_si_001.pdf [file oc3c01438_si_001.pdf]

Supplementary Information for

Seeing is Believing: Developing Multimodal Metabolic Insights at the Molecular Level

Rahuljeet S. Chadha,[1,#] Jason A. Guerrero,[2,#] Lu Wei,[1,*] Laura M Sanchez[2,*]

1. Division of Chemistry and Chemical Engineering, California Institute of Technology, Pasadena, CA, USA
2. Department of Chemistry and Biochemistry, University of California, Santa Cruz, Santa Cruz, CA, USA
# These authors contributed equally
*Corresponding authors: lmsanche@ucsc.edu, lwei@caltech.edu

Table S1. List of metabolites for stimulated Raman imaging………………………….. S2

List of Acronyms and Non-Standard Abbreviations………………………………………. S4

References……………………………………………………………………………………. S6

Table S1. List of metabolites for stimulated Raman imaging of untargeted (unlabeled) or targeted (labeled using biorthogonal tags) along with their Raman shift (cm^{-1}) and vibrational contrast.

Metabolite(s)	Targeted or Untargeted	Raman shift (cm^{-1}) and vibrational contrast
Proteins	Untargeted	2950 cm^{-1} (CH_3 stretching)[1]
Amino Acids	Targeted	2133 cm^{-1} (C–D, deuterated-AA)[2–5]<br>2123 cm^{-1} (C≡C, HPG)[6,7]
Lipids	Untargeted	2845 cm^{-1} (CH_2 stretching)[1,8]
Cholesteryl Ester (CE)	Untargeted	2957-2997 cm^{-1} (C–H of sterol rings)[9]
Triglyceride (TG)	Untargeted	3022 cm^{-1} (=C–H)[10,11]
Fatty Acids	Targeted	2110 cm^{-1} (C–D, deuterated FA)[10,12–17]<br>2250 cm^{-1} (=C–D, deuterated UFA)[10]<br>2125 cm^{-1} (C≡C, 17-ODYA)[6,7,18]
Cholesterol	Untargeted<br><br>Targeted	1669 cm^{-1} (sterol C=C)[19]<br><br>2254 cm^{-1} (C≡C, PhDY-Chol)[20]<br>2120 cm^{-1} (C–D, D_{38}-cholesterol)[21]<br>2120 cm^{-1} (alkynyl steroid)[22]
Desmosterol	Targeted	2110 cm^{-1} (C–D, D_6-desmosterol)[23]
Retinoids (Vitamin A, Retinal, Retinoic Acid)	Untargeted	1580 cm^{-1} (C=C in retinoic chain)[24]<br>1655 cm^{-1} (alkanal in retinal)[24]
Sphingomyelin (SM)	Targeted	2263 cm^{-1} (C≡C, diyne-SM)[25]
Squalene	Untargeted	1665 cm^{-1} (C=C, cis stretching)[26]
Ubiquinone (CoQ10)	Targeted	2258 cm^{-1} (C≡C, AltQ, alkyne CoQ)[27]
Vitamin E (α-Tocopherol)	Untargeted	2800-3000 cm^{-1} (C–H stretch)[28,29]
Glucose	Targeted	2129 cm^{-1} (C≡C, 3-OPG)[30,31]<br>2120 cm^{-1} (C–D, D_7-glucose)[13,14,31–34]
Nucleic Acid, DNA	Targeted	2122 cm^{-1} (C≡C, EdU)[3,6,7,35]
Nucleic Acid, RNA	Targeted	2120 cm^{-1} (C≡C, EU)[3,6]
Glycan	Targeted	2120 cm^{-1} (C≡C, Ac4ManNAl)[7]
Sucrose	Targeted	2116 cm^{-1} (C≡C, alkyne sucrose)[36]
Choline	Targeted	2188 cm^{-1} (C–D, D_9-choline)[37]<br>2142 cm^{-1} (C≡C, propargylcholine)[2,3,6]
Anisomycin, (PC)	Targeted	2219 cm^{-1} (C≡C, diyne, BADY-Ans)[38]<br>2236 cm^{-1} (C≡C, diyne, PhDY-Ans)[38]
Antimycin (PC)	Targeted	2251 cm^{-1} (C≡C, diyne, PhDY-Ant)[39]
Chloroquine (PC)	Untargeted	1370 cm^{-1} (quinoline ring stretching)[40]
Erlotinib (PC)	Targeted	2110 cm^{-1} (C≡C)[41]
Imatinib (PC)	Untargeted	1305 cm^{-1} ($C_{pyrimidine}$–$C_{pyridine}$)[40]
S-citalopram (PC)	Targeted	2170 cm^{-1} (C≡C, Alk-S-cit)[42]

Ferrostatin-1 (PC)	Targeted	2262 cm^{-1} (C≡C, diyne ferrostatin-1)[43]
Nilotinib (PC)	Untargeted	1305 cm^{-1} ($C_{pyrimidine}$–$C_{pyridine}$)[40]
Ponatinib (PC)	Targeted	2221 cm^{-1} (C≡C)[44]
Terbinafine HCl (PC)	Targeted	2230 cm^{-1} (C≡C)[6]
Acetylcholine	Untargeted	720 cm^{-1} (C–N symmetric)[45]
Fludioxonil (fungicide)	Targeted	2219 cm^{-1} (C≡N)[46]
b-carotene	Untargeted	1157 cm^{-1} (C–C) and 1526 cm^{-1} (C=C)[47,48]
Dopamine	Targeted	2135 cm^{-1} (C–D, d_4-dopamine)[49]
GABA	Targeted	2149 cm^{-1} (C–D, d_6-GABA)[49]
Propylene Glycol (PG)	Targeted	2120 cm^{-1} (C–D, d_8-PG)[50]
Water	Untargeted<br><br>Targeted	1900-2450 cm^{-1} (H–O–H bend-libration)[51]<br><br>2489 cm^{-1} (deuterated water, D–O)[52]
MitoBADY (Mitochondria)	Targeted	2220 cm^{-1} (C≡C, diyne)[53]
TPP-BDDBPDM (Mitochondria)	Targeted	2216 cm^{-1} (C≡C, diyne)[54]
Carbow2141 Mito (Mitochondria)	Targeted	2141 cm^{-1} (C≡C, polyyne)[55]
PDDA-P3 (Mitochondria)	Targeted	2120 cm^{-1} (C≡C)[56]
Carbow2141 Lyso (Lysosome)	Targeted	2141 cm^{-1} (C≡C, polyyne)[55]
PDDA-P2 (Lysosome)	Targeted	2120 cm^{-1} (C≡C)[56]
Carbow2226 ER (Endoplasmic Reticulum)	Targeted	2226 cm^{-1} (C≡C, polyyne)[55]

List of Acronyms and Non-Standard Abbreviations:

1,5-DAN: 1,5-Diaminonaphthalene
4-APEBA: (2-(4-aminophenoxy)ethyl)(4-bromophenethyl)-dimethylammonium bromide hydrobromide
9-AA: 9-Aminoacridine
AA: Amino Acid
ACN: Acetonitrile
BHp: Benzophenone
BADY: bisaryl butadiyne
CE: Cholesteryl Ester
CHCA: α-Cyano-4-hydroxycinnamic Acid
CoQ: Coenzyme Q10
DBDA: N1,N4-dibenzylidene benzene-1,4-diamine
DHB/B: 2,5-Dihydroxybenzoic Acid Butylamine Salt
DHBA: 2,5-Dihydroxybenzoic Acid
DMAN: 1,8-Bis(dimethylamino)naphthalene
EdU: 5-Ethynyl-2′-deoxyuridine
EU: 5-Ethynyl Uridine
FA: Fatty Acid
FMP-10: 4-(anthracen-9-yl)-2-fluoro-1-methylpyridin-1-ium iodide
FWHM: Full Width Half Maximum
GAMSI: Gel-Assisted Mass Spectrometry Imaging
HCl: Hydrochloride
HPG: Homopropargylglycine
hSRS: Hyperspectral SRS
IMS: Ion mobility spectrometry
IPA: Isopropanol
LD: Lipid droplet
MAGNIFIERS: Molecule Anchorable Gel-enabled Nanoscale Imaging of Fluorescence and Stimulated Raman scattering microscopy
MALDI: Matrix-assisted Laser Desorption/Ionization
MOE: Murine Oviductal Epithelial
MOSE: Murine Ovarian Surface Epithelial
MUFA: Monounsaturated Fatty Acid
NOR:9H-Pyrido[3,4-b]indole
ODYA: Octadecynoic Acid
PC: Pharmaceutical Compounds
PhDY: phenyl butadiyne
PDDA: Poly(deca-4,6-diynedioic Acid)
PG: Propylene Glycol
SCD1: Steaoryl-coA Desaturase-1
SFA: Saturated fatty acid
SM: Sphingomyelin

SRS: Stimulated Raman Scattering
TG: Triglyceride
THAP: 2',4',6'-Trihydroxyacetophenone Monohydrate
TIMS: Trapped Ion Mobility
TPP-BDDBPDM: Triphenylphosphonium Buta-1,3-diyne-1,4-diylbis(4,1-phenylene))dimethanol
UFA: Unsaturated Fatty Acid
VISTA: Vibrational Imaging of Swelled Tissues and Analysis

References

(1) Saar, B. G.; Freudiger, C. W.; Reichman, J.; Stanley, C. M.; Holtom, G. R.; Xie, X. S. Video-Rate Molecular Imaging in Vivo with Stimulated Raman Scattering. *Science* **2010**, *330* (6009), 1368–1370.

(2) Zhang, L.; Min, W. Bioorthogonal Chemical Imaging of Metabolic Changes during Epithelial-Mesenchymal Transition of Cancer Cells by Stimulated Raman Scattering Microscopy. *J. Biomed. Opt.* **2017**, *22* (10), 1–7.

(3) Hu, F.; Lamprecht, M. R.; Wei, L.; Morrison, B.; Min, W. Bioorthogonal Chemical Imaging of Metabolic Activities in Live Mammalian Hippocampal Tissues with Stimulated Raman Scattering. *Sci. Rep.* **2016**, *6*, 39660.

(4) Wei, L.; Shen, Y.; Xu, F.; Hu, F.; Harrington, J. K.; Targoff, K. L.; Min, W. Imaging Complex Protein Metabolism in Live Organisms by Stimulated Raman Scattering Microscopy with Isotope Labeling. *ACS Chem. Biol.* **2015**, *10* (3), 901–908.

(5) Wei, L.; Yu, Y.; Shen, Y.; Wang, M. C.; Min, W. Vibrational Imaging of Newly Synthesized Proteins in Live Cells by Stimulated Raman Scattering Microscopy. *Proc. Natl. Acad. Sci. U. S. A.* **2013**, *110* (28), 11226–11231.

(6) Wei, L.; Hu, F.; Shen, Y.; Chen, Z.; Yu, Y.; Lin, C.-C.; Wang, M. C.; Min, W. Live-Cell Imaging of Alkyne-Tagged Small Biomolecules by Stimulated Raman Scattering. *Nat. Methods* **2014**, *11* (4), 410–412.

(7) Hong, S.; Chen, T.; Zhu, Y.; Li, A.; Huang, Y.; Chen, X. Live-Cell Stimulated Raman Scattering Imaging of Alkyne-Tagged Biomolecules. *Angew. Chem. Int. Ed Engl.* **2014**, *53* (23), 5827–5831.

(8) Freudiger, C. W.; Min, W.; Saar, B. G.; Lu, S.; Holtom, G. R.; He, C.; Tsai, J. C.; Kang, J. X.; Xie, X. S. Label-Free Biomedical Imaging with High Sensitivity by Stimulated Raman Scattering Microscopy. *Science* **2008**, *322* (5909), 1857–1861.

(9) Yue, S.; Li, J.; Lee, S.-Y.; Lee, H. J.; Shao, T.; Song, B.; Cheng, L.; Masterson, T. A.; Liu, X.; Ratliff, T. L.; Cheng, J.-X. Cholesteryl Ester Accumulation Induced by PTEN Loss and PI3K/AKT Activation Underlies Human Prostate Cancer Aggressiveness. *Cell Metab.* **2014**, *19* (3), 393–406.

(10) Fu, D.; Yu, Y.; Folick, A.; Currie, E.; Farese, R. V., Jr; Tsai, T.-H.; Xie, X. S.; Wang, M. C. In Vivo Metabolic Fingerprinting of Neutral Lipids with Hyperspectral Stimulated Raman Scattering Microscopy. *J. Am. Chem. Soc.* **2014**, *136* (24), 8820–8828.

(11) Li, J.; Condello, S.; Thomes-Pepin, J.; Ma, X.; Xia, Y.; Hurley, T. D.; Matei, D.; Cheng, J.-X. Lipid Desaturation Is a Metabolic Marker and Therapeutic Target of Ovarian Cancer Stem Cells. *Cell Stem Cell* **2017**, *20* (3), 303–314.e5.

(12) Li, X.; Li, Y.; Jiang, M.; Wu, W.; He, S.; Chen, C.; Qin, Z.; Tang, B. Z.; Mak, H. Y.; Qu, J. Y. Quantitative Imaging of Lipid Synthesis and Lipolysis Dynamics in Caenorhabditis Elegans by Stimulated Raman Scattering Microscopy. *Anal. Chem.* **2019**, *91* (3), 2279–2287.

(13) Tan, Y.; Li, J.; Zhao, G.; Huang, K.-C.; Cardenas, H.; Wang, Y.; Matei, D.; Cheng, J.-X. Metabolic Reprogramming from Glycolysis to Fatty Acid Uptake and Beta-Oxidation in Platinum-Resistant Cancer Cells. *Nat. Commun.* **2022**, *13* (1), 4554.

(14) Li, J.; Cheng, J.-X. Direct Visualization of de Novo Lipogenesis in Single Living Cells. *Sci. Rep.* **2014**, *4*, 6807.

(15) Yu, Y.; Mutlu, A. S.; Liu, H.; Wang, M. C. High-Throughput Screens Using Photo-Highlighting Discover BMP Signaling in Mitochondrial Lipid Oxidation. *Nat. Commun.* **2017**, *8* (1), 865.

(16) Stiebing, C.; Meyer, T.; Rimke, I.; Matthäus, C.; Schmitt, M.; Lorkowski, S.; Popp, J. Real-Time Raman and SRS Imaging of Living Human Macrophages Reveals Cell-to-Cell Heterogeneity and Dynamics of Lipid Uptake. *J. Biophotonics* **2017**, *10* (9), 1217–1226.

(17) Matthäus, C.; Krafft, C.; Dietzek, B.; Brehm, B. R.; Lorkowski, S.; Popp, J. Noninvasive

Imaging of Intracellular Lipid Metabolism in Macrophages by Raman Microscopy in Combination with Stable Isotopic Labeling. *Anal. Chem.* **2012**, *84* (20), 8549–8556.
(18) Chen, Z.; Paley, D. W.; Wei, L.; Weisman, A. L.; Friesner, R. A.; Nuckolls, C.; Min, W. Multicolor Live-Cell Chemical Imaging by Isotopically Edited Alkyne Vibrational Palette. *J. Am. Chem. Soc.* **2014**, *136* (22), 8027–8033.
(19) Wang, P.; Li, J.; Wang, P.; Hu, C.-R.; Zhang, D.; Sturek, M.; Cheng, J.-X. Label-Free Quantitative Imaging of Cholesterol in Intact Tissues by Hyperspectral Stimulated Raman Scattering Microscopy. *Angew. Chem. Int. Ed Engl.* **2013**, *52* (49), 13042–13046.
(20) Lee, H. J.; Zhang, W.; Zhang, D.; Yang, Y.; Liu, B.; Barker, E. L.; Buhman, K. K.; Slipchenko, L. V.; Dai, M.; Cheng, J.-X. Assessing Cholesterol Storage in Live Cells and C. Elegans by Stimulated Raman Scattering Imaging of Phenyl-Diyne Cholesterol. *Sci. Rep.* **2015**, *5*, 7930.
(21) Alfonso-García, A.; Pfisterer, S. G.; Riezman, H.; Ikonen, E.; Potma, E. O. D38-Cholesterol as a Raman Active Probe for Imaging Intracellular Cholesterol Storage. *J. Biomed. Opt.* **2016**, *21* (6), 61003.
(22) Yamaguchi, S.; Matsushita, T.; Izuta, S.; Katada, S.; Ura, M.; Ikeda, T.; Hayashi, G.; Suzuki, Y.; Kobayashi, K.; Tokunaga, K.; Ozeki, Y.; Okamoto, A. Chemically-Activatable Alkyne-Tagged Probe for Imaging Microdomains in Lipid Bilayer Membranes. *Sci. Rep.* **2017**, *7*, 41007.
(23) Villareal, V. A.; Fu, D.; Costello, D. A.; Xie, X. S.; Yang, P. L. Hepatitis C Virus Selectively Alters the Intracellular Localization of Desmosterol. *ACS Chem. Biol.* **2016**, *11* (7), 1827–1833.
(24) Chen, A. J.; Li, J.; Jannasch, A.; Mutlu, A. S.; Wang, M. C.; Cheng, J.-X. Fingerprint Stimulated Raman Scattering Imaging Reveals Retinoid Coupling Lipid Metabolism and Survival. *Chemphyschem* **2018**, *19* (19), 2500–2506.
(25) Cui, J.; Matsuoka, S.; Kinoshita, M.; Matsumori, N.; Sato, F.; Murata, M.; Ando, J.; Yamakoshi, H.; Dodo, K.; Sodeoka, M. Novel Raman-Tagged Sphingomyelin That Closely Mimics Original Raft-Forming Behavior. *Bioorg. Med. Chem.* **2015**, *23* (13), 2989–2994.
(26) Ishitsuka, K.; Koide, M.; Yoshida, M.; Segawa, H.; Leproux, P.; Couderc, V.; Watanabe, M. M.; Kano, H. Identification of Intracellular Squalene in Living algae,Aurantiochytrium Mangroveiwith Hyper-Spectral Coherent Anti-Stokes Raman Microscopy Using a Sub-Nanosecond Supercontinuum Laser Source. *J. Raman Spectrosc.* **2017**, *48* (1), 8–15.
(27) Yamakoshi, H.; Dodo, K.; Palonpon, A.; Ando, J.; Fujita, K.; Kawata, S.; Sodeoka, M. Alkyne-Tag Raman Imaging for Visualization of Mobile Small Molecules in Live Cells. *J. Am. Chem. Soc.* **2012**, *134* (51), 20681–20689.
(28) Liao, C.-S.; Wang, P.; Wang, P.; Li, J.; Lee, H. J.; Eakins, G.; Cheng, J.-X. Spectrometer-Free Vibrational Imaging by Retrieving Stimulated Raman Signal from Highly Scattered Photons. *Sci Adv* **2015**, *1* (9), e1500738.
(29) Beattie, J. R.; Maguire, C.; Gilchrist, S.; Barrett, L. J.; Cross, C. E.; Possmayer, F.; Ennis, M.; Elborn, J. S.; Curry, W. J.; McGarvey, J. J.; Schock, B. C. The Use of Raman Microscopy to Determine and Localize Vitamin E in Biological Samples. *FASEB J.* **2007**, *21* (3), 766–776.
(30) Hu, F.; Chen, Z.; Zhang, L.; Shen, Y.; Wei, L.; Min, W. Vibrational Imaging of Glucose Uptake Activity in Live Cells and Tissues by Stimulated Raman Scattering. *Angew. Chem. Int. Ed Engl.* **2015**, *54* (34), 9821–9825.
(31) Long, R.; Zhang, L.; Shi, L.; Shen, Y.; Hu, F.; Zeng, C.; Min, W. Two-Color Vibrational Imaging of Glucose Metabolism Using Stimulated Raman Scattering. *Chem. Commun.* **2018**, *54* (2), 152–155.
(32) Du, J.; Su, Y.; Qian, C.; Yuan, D.; Miao, K.; Lee, D.; Ng, A. H. C.; Wijker, R. S.; Ribas, A.; Levine, R. D.; Heath, J. R.; Wei, L. Raman-Guided Subcellular Pharmaco-Metabolomics for Metastatic Melanoma Cells. *Nat. Commun.* **2020**, *11* (1), 4830.

(33) Lee, D.; Du, J.; Yu, R.; Su, Y.; Heath, J. R.; Wei, L. Visualizing Subcellular Enrichment of Glycogen in Live Cancer Cells by Stimulated Raman Scattering. *Anal. Chem.* **2020**, *92* (19), 13182–13191.
(34) Zhang, L.; Shi, L.; Shen, Y.; Miao, Y.; Wei, M.; Qian, N.; Liu, Y.; Min, W. Spectral Tracing of Deuterium for Imaging Glucose Metabolism. *Nat Biomed Eng* **2019**, *3* (5), 402–413.
(35) Yamakoshi, H.; Dodo, K.; Okada, M.; Ando, J.; Palonpon, A.; Fujita, K.; Kawata, S.; Sodeoka, M. Imaging of EdU, an Alkyne-Tagged Cell Proliferation Probe, by Raman Microscopy. *J. Am. Chem. Soc.* **2011**, *133* (16), 6102–6105.
(36) de Moliner, F.; Knox, K.; Gordon, D.; Lee, M.; Tipping, W. J.; Geddis, A.; Reinders, A.; Ward, J. M.; Oparka, K.; Vendrell, M. A Palette of Minimally Tagged Sucrose Analogues for Real-Time Raman Imaging of Intracellular Plant Metabolism. *Angew. Chem. Int. Ed Engl.* **2021**, *60* (14), 7637–7642.
(37) Hu, F.; Wei, L.; Zheng, C.; Shen, Y.; Min, W. Live-Cell Vibrational Imaging of Choline Metabolites by Stimulated Raman Scattering Coupled with Isotope-Based Metabolic Labeling. *Analyst* **2014**, *139* (10), 2312–2317.
(38) Tipping, W. J.; Lee, M.; Serrels, A.; Brunton, V. G.; Hulme, A. N. Imaging Drug Uptake by Bioorthogonal Stimulated Raman Scattering Microscopy. *Chem. Sci.* **2017**, *8* (8), 5606–5615.
(39) Seidel, J.; Miao, Y.; Porterfield, W.; Cai, W.; Zhu, X.; Kim, S.-J.; Hu, F.; Bhattarai-Kline, S.; Min, W.; Zhang, W. Structure-Activity-Distribution Relationship Study of Anti-Cancer Antimycin-Type Depsipeptides. *Chem. Commun.* **2019**, *55* (63), 9379–9382.
(40) Fu, D.; Zhou, J.; Zhu, W. S.; Manley, P. W.; Wang, Y. K.; Hood, T.; Wylie, A.; Xie, X. S. Imaging the Intracellular Distribution of Tyrosine Kinase Inhibitors in Living Cells with Quantitative Hyperspectral Stimulated Raman Scattering. *Nat. Chem.* **2014**, *6* (7), 614–622.
(41) El-Mashtoly, S. F.; Petersen, D.; Yosef, H. K.; Mosig, A.; Reinacher-Schick, A.; Kötting, C.; Gerwert, K. Label-Free Imaging of Drug Distribution and Metabolism in Colon Cancer Cells by Raman Microscopy. *Analyst* **2014**, *139* (5), 1155–1161.
(42) Tanuma, M.; Kasai, A.; Bando, K.; Kotoku, N.; Harada, K.; Minoshima, M.; Higashino, K.; Kimishima, A.; Arai, M.; Ago, Y.; Seiriki, K.; Kikuchi, K.; Kawata, S.; Fujita, K.; Hashimoto, H. Direct Visualization of an Antidepressant Analog Using Surface-Enhanced Raman Scattering in the Brain. *JCI Insight* **2020**, *5* (6). https://doi.org/10.1172/jci.insight.133348.
(43) Gaschler, M. M.; Hu, F.; Feng, H.; Linkermann, A.; Min, W.; Stockwell, B. R. Determination of the Subcellular Localization and Mechanism of Action of Ferrostatins in Suppressing Ferroptosis. *ACS Chem. Biol.* **2018**, *13* (4), 1013–1020.
(44) Sepp, K.; Lee, M.; Bluntzer, M. T. J.; Helgason, G. V.; Hulme, A. N.; Brunton, V. G. Utilizing Stimulated Raman Scattering Microscopy To Study Intracellular Distribution of Label-Free Ponatinib in Live Cells. *J. Med. Chem.* **2020**, *63* (5), 2028–2034.
(45) Fu, D.; Yang, W.; Xie, X. S. Label-Free Imaging of Neurotransmitter Acetylcholine at Neuromuscular Junctions with Stimulated Raman Scattering. *J. Am. Chem. Soc.* **2017**, *139* (2), 583–586.
(46) Wang, C.-C.; Moorhouse, S.; Stain, C.; Seymour, M.; Green, E.; Penfield, S.; Moger, J. In Situ Chemically Specific Mapping of Agrochemical Seed Coatings Using Stimulated Raman Scattering Microscopy. *J. Biophotonics* **2018**, *11* (11), e201800108.
(47) You, A. Y. F.; Bergholt, M. S.; St-Pierre, J.-P.; Kit-Anan, W.; Pence, I. J.; Chester, A. H.; Yacoub, M. H.; Bertazzo, S.; Stevens, M. M. Raman Spectroscopy Imaging Reveals Interplay between Atherosclerosis and Medial Calcification in the Human Aorta. *Sci Adv* **2017**, *3* (12), e1701156.
(48) Buschman, H. P.; Deinum, G.; Motz, J. T.; Fitzmaurice, M.; Kramer, J. R.; van der Laarse, A.; Bruschke, A. V.; Feld, M. S. Raman Microspectroscopy of Human Coronary Atherosclerosis: Biochemical Assessment of Cellular and Extracellular Morphologic

Structures in Situ. *Cardiovasc. Pathol.* **2001**, *10* (2), 69–82.
(49) Dorlhiac, G. F.; Landry, M. P.; Streets, A. Leveraging Isotopologues as a General Strategy to Image Neurotransmitters with Vibrational Microscopy. *arXiv [physics.bio-ph]*, 2022. https://doi.org/10.48550/ARXIV.2205.05798.
(50) Chiu, W. S.; Belsey, N. A.; Garrett, N. L.; Moger, J.; Delgado-Charro, M. B.; Guy, R. H. Molecular Diffusion in the Human Nail Measured by Stimulated Raman Scattering Microscopy. *Proc. Natl. Acad. Sci. U. S. A.* **2015**, *112* (25), 7725–7730.
(51) Ramos, S.; Lee, J. C. Water Bend-Libration as a Cellular Raman Imaging Probe of Hydration. *Proc. Natl. Acad. Sci. U. S. A.* **2023**, *120* (42), e2313133120.
(52) Shi, L.; Zheng, C.; Shen, Y.; Chen, Z.; Silveira, E. S.; Zhang, L.; Wei, M.; Liu, C.; de Sena-Tomas, C.; Targoff, K.; Min, W. Optical Imaging of Metabolic Dynamics in Animals. *Nat. Commun.* **2018**, *9* (1), 2995.
(53) Yamakoshi, H.; Palonpon, A.; Dodo, K.; Ando, J.; Kawata, S.; Fujita, K.; Sodeoka, M. A Sensitive and Specific Raman Probe Based on Bisarylbutadiyne for Live Cell Imaging of Mitochondria. *Bioorg. Med. Chem. Lett.* **2015**, *25* (3), 664–667.
(54) Bae, K.; Zheng, W.; Ma, Y.; Huang, Z. Real-Time Monitoring of Pharmacokinetics of Mitochondria-Targeting Molecules in Live Cells with Bioorthogonal Hyperspectral Stimulated Raman Scattering Microscopy. *Anal. Chem.* **2020**, *92* (1), 740–748.
(55) Hu, F.; Zeng, C.; Long, R.; Miao, Y.; Wei, L.; Xu, Q.; Min, W. Supermultiplexed Optical Imaging and Barcoding with Engineered Polyynes. *Nat. Methods* **2018**, *15* (3), 194–200.
(56) Tian, S.; Li, H.; Li, Z.; Tang, H.; Yin, M.; Chen, Y.; Wang, S.; Gao, Y.; Yang, X.; Meng, F.; Lauher, J. W.; Wang, P.; Luo, L. Polydiacetylene-Based Ultrastrong Bioorthogonal Raman Probes for Targeted Live-Cell Raman Imaging. *Nat. Commun.* **2020**, *11* (1), 81.
