# Supplementary material for: Seeing is Believing: Developing Multimodal Metabolic Insights at the Molecular Level
Source: ACS Cent Sci. 2024 Mar 21;10(4):758–74. doi: 10.1021/acscentsci.3c01438 (PMC11046475; doi:10.1021/acscentsci.3c01438)
Supplement: Supplementary file 2 — oc3c01438_si_002.pdf [file oc3c01438_si_002.pdf]

oc-2023-01438b.R1

Name: Peer Review Information for "Seeing is Believing: Developing Multimodal Metabolic Insights at the Molecular Level"

First Round of Reviewer Comments

Reviewer: 1

Comments to the Author

The authors present a review of the literature discussing the utility of multi-modal imaging combining mass spectrometry imaging and stimulated Raman spectroscopy. The review givers users both background on the techniques and cite relevant current literature. Minor comments are provided below to help enhance the manuscript further.

- For figure 1A, I would try and make some denotation of the separations in the portions of the schematic. The figure is a bit busy and makes it more difficult to interpret.

- Page 6, line 56, you discuss crystal sizes, I would make sure that the reader understands that this is only the case for matrix sublimation/spray early on.

-Page 7, line 3, you discuss that the laser spot size of the MALDI is 5-10 um. While this is true for the timsTOF (5um) system, the medium-large settings found on older Bruker instruments (FT-ICR/Ultraflex) have larger ablation craters.

-Page 7 line 6, word choice should be "lengthy" not "length"

-Figure 2 is formatted odd with the end image being at the top of the figure (part d). While not wrong, I would flip the figure to have the beginning steps starting at the top.

-For figure 2's caption, denote that samples are placed on "conductive surfaces to encompass both the use of target plates and ITO slides

-The description of resolution power and its necessity for MSI experiments may fit better into the main body of the work rather than as a figure caption that could be glossed over by readers.

-Page 9, line 11, the sentence has two ands together which make it hard to read, potentially consider a semi-colon or splitting into two sentences.

-Table 1, Line F, add in that 2,5-dihydroxybenzoic acid is also denoted as DHB since it is used that way in the article. For line J, you may wish to include the name "Norharmane" as well.

-Page 15, Line 15 MALDI is spelled incorrectly

-Figure 4 when zoomed in gets slightly blurry on my screen. If possible I would try to fix.

-Make sure to update any rxiv-based references. For example, Zemaitis et al. [67] was published outside of Rxiv's in August 2023.

Overall this was a highly interesting read and I believe it will be of great interest to the analytical chemistry community.

Author's Response to Peer Review Comments:

Reviewer(s)' Comments to Author:

The authors present a review of the literature discussing the utility of multi-modal imaging combining mass spectrometry imaging and stimulated Raman spectroscopy. The review gives users both background on the techniques and cites relevant current literature. Minor comments are provided below to help enhance the manuscript further.

1. For ***figure 1A***, I would try and make some denotation of the separations in the portions of the schematic. The figure is a bit busy and makes it more difficult to interpret.

   We thank the reviewer for their suggestion. We have further simplified Figure 1A.

2. ***Page 6, line 56,*** you discuss crystal sizes, I would make sure that the reader understands that this is only the case for matrix sublimation/spray early on.

   We have amended this sentence to now read as follows:

   The spatial resolution is limited for a number of reasons, including the size of the matrix crystals created via sublimation or spraying (<5µm),[53,54] but also by the stage movement (raster step) and spot size of the laser (5µm-10µm).[55]

3. ***Page 7, line 3***, you discuss that the laser spot size of the MALDI is 5-10 um. While this is true for the timsTOF (5um) system, the medium-large settings found on older Bruker instruments (FT-ICR/Ultraflex) have larger ablation craters.

   This is an excellent point raised by the reviewer, we have clarified this sentence further as follows:

   The spatial resolution is limited for a number of reasons, including the size of the matrix crystals created via sublimation or spraying (<5µm),[53,54] but also by the stage movement (raster step) and spot size of the laser (5µm-10µm).[55] It is worth noting that these

numbers can be achieved with newer commercial mass spectrometers, while older instrumentation, which still has value and are widely used will have lower spatial resolution capabilities.

4. ***Page 7 line 6,*** word choice should be "lengthy" not "length"

   We thank the reviewer for catching this typo, we have changed the text from “length” to “lengthy.”

5. ***Figure 2*** is formatted odd with the end image being at the top of the figure (part d). While not wrong, I would flip the figure to have the beginning steps starting at the top.

   We apologize for this confusion, we have amended this figure now by flipping the image, positioning the target plate with the sample at the top and the 2D ion image at the bottom. The initial draft of the image was created to illustrate the ablated surface, showcasing ions and matrix ascending through a vacuum to acquire an overall average mass spectra. We were attempting to follow a linear flight path if you will, but this nuance likely led to more confusion.

6. For ***figure 2's*** caption, denote that samples are placed on "conductive surfaces to encompass both the use of target plates and ITO slides

   Figure 2A caption has been amended to read as follows:

   Adherence typically begins by placing a fresh frozen thin tissue section onto a conductive surface such as a target plate (as depicted), where it is thaw mounted and thus adhered to the plate. Samples that are grown in or on agar or agarose can be dried directly onto the conductive surface. In instances of charged decoupled sources, conductive surfaces are not needed.

7. The description of resolution power and its necessity for MSI experiments may fit better into the main body of the work rather than as a figure caption that could be glossed over by readers.

   We agree with this reviewer and have moved the figure caption to the main body of the text and have simplified the resulting figure capture.

8. ***Page 9, line 11,*** the sentence has two ands together which make it hard to read, potentially consider a semi-colon or splitting into two sentences.

   We thank the reviewer for this suggestion and have incorporated a semicolon rather than a second ‘and.’

9. ***Table 1, Line F,*** add in that 2,5-dihydroxybenzoic acid is also denoted as DHB since it is used that way in the article. For line J, you may wish to include the name "Norharmane" as well.

   We thank the reviewer for this suggestion, both “F” and “J” have been adjusted as recommended.

10. ***Page 15***, Line 15 MALDI is spelled incorrectly

    We thank the reviewer for highlighting the error, we have corrected the spelling in the revised manuscript.

11. ***Figure 4*** when zoomed in gets slightly blurry on my screen. If possible I would try to fix.

    Figure 4 has been refined to eliminate any perceived blurriness.

12. Make sure to update any rxiv-based references. For example, Zemaitis et al. [67] was published outside of Rxiv's in August 2023.

    We have updated the Rxiv based references when possible. References 53, 65, 108, 125 are still preprints. Whereas references 67 and 158 have been updated as they are peer reviewed manuscripts now.

Formatting Needs:

TOC: Please move to the last page of the manuscript, beneath the References, and label "TOC Graphic."

We have moved the TOC graphic to the last page of the manuscript, beneath the References section.

SI PARAGRAPH: If the manuscript is accompanied by any supporting information for publication, a brief description of the supplementary material is required in the manuscript. The appropriate format is: Supporting Information. Brief statement in non-sentence format listing the contents of the material supplied as Supporting Information.

We have included a description of the contents for the supporting information.

GENERAL REF FORMATTING: Periodical references should contain authors’ surnames followed by initials, article title, journal abbreviation, year, volume number, and page range. Refs with more than 10 authors should list the first 10 and then be followed by “et al.”

Web sources must include access date.

We have incorporated these changes and formatted the references accordingly.

SYNOPSIS MISSING: The synopsis should be no more than 200 characters (including spaces) and should reasonably correlate with the TOC graphic. The synopsis is intended to explain the importance of the article to a broader readership across the sciences. Please place your synopsis in the manuscript file after the TOC graphic, and label it as "Synopsis."

We have added a Synopsis section and labeled it as such at the end of the manuscript.

CAPTION PERMISSIONS: One or more of the figures/tables/schemes include reference notification. Please confirm that this pertains to data used, and not the image itself.

If any of your graphics have been previously published this should be stated in the caption and copyright permissions should be obtained from any non ACS publications. Upload copies of the permissions as Other Files for Editors Only with your revision. Please note: If you have permitted use as the author of the previously published graphics, you do not need to supply written copyright permission.

*We have permitted use as the author of the previously published graphics in **Figure 4** (Figure adapted from Ref 45) and **Figure 5** (Figure adapted from Ref 152).*

Credit lines should read as follows: "Reproduced [or Adapted, Reprinted, etc.] with permission from Ref. [number], Copyright [year] [journal name]."Please visit for more information https://pubs.acs.org/page/copyright/index.html

All credit lines should now follow the recommended format.

PULL QUOTE (OUTLOOKS + IN FOCUS): We encourage you to select 3 - 4 quotes from your submission that you would like highlighted in your paper. The quotes should be one sentencelong, unique to the submission and not from previously cited work. Please list your quotes at the end of the manuscript file.

We have selected 5 pull quotes for consideration and they are labeled as such at the end of the manuscript.
